# The role of indole‐3‐acetic acid and characterization of PIN transporters in complex streptophyte alga *Chara braunii*

**DOI:** 10.1111/nph.70019

**Published:** 2025-03-06

**Authors:** Katarina Kurtović, Stanislav Vosolsobě, Daniel Nedvěd, Karel Müller, Petre Ivanov Dobrev, Vojtěch Schmidt, Piotr Piszczek, Andre Kuhn, Adrijana Smoljan, Tom J. Fisher, Dolf Weijers, Jiří Friml, John L. Bowman, Jan Petrášek

**Affiliations:** ^1^ Department of Experimental Plant Biology, Faculty of Science Charles University Viničná 5 Prague 2 128 44 Czech Republic; ^2^ Laboratory of Hormonal Regulations in Plants Institute of Experimental Botany of the Czech Academy of Sciences Rozvojová 263 Prague 6 165 02 Czech Republic; ^3^ Faculty of Biotechnology University of Wroclaw Joliot‐Curie 14a Wroclaw 50‐383 Poland; ^4^ Laboratory of Biochemistry Wageningen University Stippeneng 4 Wageningen 6708 SP the Netherlands; ^5^ Department of Plant Cell Biology, Green Life Sciences Cluster, Swammerdam Institute for Life Sciences University of Amsterdam Amsterdam 1098XH the Netherlands; ^6^ Institute of Science and Technology Austria (ISTA) Klosterneuburg 3400 Austria; ^7^ School of Biological Sciences Monash University Melbourne 3800 Vic. Australia; ^8^ ARC Centre of Excellence for Plant Success in Nature and Agriculture Monash University Melbourne 3800 Vic. Australia

**Keywords:** auxin transport, *Chara*, indole‐3‐acetic acid, plant evolution, streptophytes

## Abstract

- Auxin, indole‐3‐acetic acid (IAA), is a key phytohormone with diverse morphogenic roles in land plants, but its function and transport mechanisms in algae remain poorly understood. We therefore aimed to explore the role of IAA in a complex, streptophyte algae *Chara braunii*.
- Here, we described novel responses of *C. braunii* to IAA and characterized two homologs of PIN auxin efflux carriers: CbPINa and CbPINc. We determined their localization in *C. braunii* using epitope‐specific antibodies and tested their function in heterologous land plant models. Further, using phosphoproteomic analysis, we identified IAA‐induced phosphorylation events.
- The thallus regeneration assay showed that IAA promotes thallus elongation and side branch development. Immunolocalization of CbPINa and CbPINc confirmed their presence on the plasma membrane of vegetative and generative cells of *C. braunii.* However, functional assays in tobacco BY‐2 cells demonstrated that CbPINa affects auxin transport, whereas CbPINc does not. The IAA is effective in the acceleration of cytoplasmic streaming and the phosphorylation of evolutionary conserved targets such as homolog of RAF‐like kinase.
- These findings suggest that, although canonical PIN‐mediated auxin transport mechanisms might not be fully conserved in *Chara*, IAA is involved in morphogenesis and fast signaling processes.

## Introduction

During the transition from water to land, plants underwent a series of developmental innovations, leading to the establishment of a complex body (Harrison, 2017; Donoghue *et al*., 2021; Bowman, 2022). A key regulator of land plant development is the phytohormone auxin, which acts through local biosynthesis and directional transport, resulting in the formation of concentration gradients (Friml, 2022). The directional flow of auxin, facilitated by the PIN family of auxin efflux carriers, is critical for plant morphogenesis and adaptive growth responses to environmental cues (Gao *et al*., 2008; Luschnig & Friml, 2024). Recent research has provided significant insights into the mechanisms of auxin action (Kuhn *et al*., 2024), including the structural elucidation of three PIN auxin efflux carriers (Su *et al*., 2022; Ung *et al*., 2022; Yang *et al*., 2022). However, the question of how and when auxin became a pivotal driver of morphological changes in land plants remains largely unanswered. This question cannot be fully addressed by studying only land plants but requires a comprehensive investigation of their algal relatives (Skokan *et al*., 2019). Land plants (embryophytes) and streptophyte green algae, from which they emerged, together form the group Streptophyta (Becker & Marin, 2009). Streptophyte algae comprise six clades, exhibiting significant morphological diversity within these clades (Buschmann, 2020; Bierenbroodspot *et al*., 2024). Among these six clades, *Chara* spp. and *Nitella* spp., members of the Charophyceae family, possess the highest degree of complexity with respect to their body plan. Due to its large internodal cells, transparent gravitropic rhizoids, and rapid cytoplasmic streaming, *Chara* has been a model organism for decades, facilitating the study of fundamental cell biological processes (Kurtović *et al*., 2024).

Although genome sequencing revealed that *Chara braunii* lacks the auxin biosynthetic pathway involving the *TAA* and *YUCCA* genes, which converts tryptophan to an auxin, indole‐3‐acetic acid (IAA) (Nishiyama *et al*., 2018), earlier and recent studies are consistently confirming the presence of IAA in the biomass of various species of *Chara* and *Nitella* (Jahnke & Libbert, 1964; Sztein *et al*., 2000; Hackenberg & Pandey, 2014; Beilby *et al*., 2015; Schmidt *et al*., 2024), suggesting the presence of an alternative biosynthetic pathway. Besides IAA, other phytohormones have been identified in the biomass of *C. braunii*, including cytokinin *N* ^6^‐(∆^2^‐isopentenyl)‐adenine, ethylene, and jasmonic acid (Schmidt *et al*., 2024). However, much less is known about their effects or role in *Chara*, and therefore, they are not the focus of this study. On the other hand, auxin transport has been implicated in *Chara* in several studies. Dibb‐Fuller & Morris (1992) demonstrated auxin influx and efflux in *Chara* cells, using radio‐labeled IAA. In addition, they showed that efflux was unaffected by the PIN‐dependent auxin transport inhibitor N‐1‐naphthylphthalamic acid (NPA) (Abas *et al*., 2021). By contrast, Boot *et al*. (2012) found NPA‐sensitive polar auxin transport in *Chara corallina*. Later, PIN‐like proteins were detected by immunolocalization using heterologous anti‐AtPIN2 antibodies in the antheridial filaments of *Chara vulgaris* (Żabka *et al*., 2016). Finally, genome sequencing confirmed that *Chara* indeed possesses six homologs of PIN auxin efflux carriers (Nishiyama *et al*., 2018), the highest number among all streptophyte algae (Hori *et al*., 2014; Cheng *et al*., 2019; Liang *et al*., 2020; Vosolsobě *et al*., 2020), at least five ATP‐binding cassette B (ABCB) homologs, but no AUX/LAX influx carriers (Nishiyama *et al*., 2018). Furthermore, exogenously applied IAA has been shown to promote rhizoid growth in decapitated *Chara* thalli (Klämbt *et al*., 1992), affect ion transport (S. Zhang *et al*., 2016), induce transient depolymerization of microtubules (Jin *et al*., 2008), and accelerate the process of differentiation of antheridial filament cells (Godlewski, 1980). However, none of these studies further explored potential modes of IAA perception by *Chara*. The genome of *Chara* encodes certain elements of the auxin signaling pathway, in particular, an AUXIN RESPONSE FACTOR (ARF) and two Aux/IAA sequences; however, these components are not functionally equivalent to those in the canonical auxin signaling pathway of land plnts (Mutte *et al*., 2018). The lack of a canonical signaling pathway can be further supported by the absence of the canonical TIR1 receptor (Mutte *et al*., 2018; Nishiyama *et al*., 2018; Bowman *et al*., 2021). On the other hand, *Chara* encodes a single homolog (Carrillo‐Carrasco *et al*., 2023) of a cell surface auxin receptor Auxin Binding Protein1 (ABP1) (Friml *et al*., 2022), suggesting the presence of a noncanonical signaling pathway (Kuhn *et al*., 2024).

Given the morphological complexity of *Chara*, the number of PIN homologs in the *Chara* genome, and the ambiguous results in literature, this study focuses on describing auxin responses and functional evaluation of *Chara* PIN homologs. We show that IAA treatments promoted side branching of regenerated thalli upon decapitation. To test the possible involvement of carrier‐mediated IAA transport in the *Chara* growth responses, we cloned two of six PIN homologs and showed that in *Nicotiana tabaccum*, BY‐2 cells CbPINa, but not CbPINc, influences the accumulation of radioactively labeled auxin. Immunolocalization using specific antibodies showed that both CbPINa and CbPINc are associated with the plasma membrane (PM) in vegetative and generative cells of *Chara*. However, their expressions in *Arabidopsis thaliana*, and bryophyte *Marchantia polymorpha*, did not rescue the mutant phenotypes, despite their association with the PM and polar localization of CbPINa in gametangiophore stalks of *Marchantia*. To analyze rapid auxin action in *Chara*, we integrated phosphoproteomic analysis with cytoplasmic streaming assay, revealing IAA‐specific changes in the phosphoproteome. We identified MAP4K homolog as a dominant target of IAA response and also the activation of a RAF‐like kinase homolog. This observation provides evidence of conserved rapid auxin signaling in streptophytes, as reported by Kuhn *et al*. (2024). Additionally, our study demonstrates that IAA promotes fast cytoplasmic streaming in branchlet internodal cells, suggesting a link between auxin‐triggered phosphorylation events and cytoplasmic streaming dynamics.

## Materials and Methods

### Plant materials and growth conditions

Two strains of *Chara braunii* C.C.Gmelin 1826, were used in this study: S276 from MAdLand (https://madland.science/) and NIES 1604 from the NIES collection (https://mcc.nies.go.jp/). Both strains were grown on modified soil‐sand medium (SWCN‐4) (https://mcc.nies.go.jp/medium/en/swcn4.pdf). In short, 1 cm of garden soil was added to the glass tube and dampened slightly with a few drops of distilled water. On top of that layer, 4–5 cm of silica sand was added, autoclaved 2×, and then supplemented with sterile distilled water. The strain NIES 1604 was used for regeneration, germination, and metabolic profiling, while strain S276 was used for DNA and RNA isolation and phosphoproteomic analysis. Cultures were maintained under fluorescent light (F15W/T8 AQUASTAR, cat no. 0002224; Sylvania) at room temperature, while regeneration experiments were conducted in a custom box with LED strips (construction of the box is detailed in Supporting Information Methods S1; Fig. S1), at 19–22°C, and a 14 h : 10 h, light : dark cycle. To germinate oospores, mature oospores were harvested, air‐dried for 1 month, and stratified at 4°C for at least 4 months to break the dormancy. Before germination, oospores were washed with 1% Tween 20, followed by disinfection with 10% commercial bleach for 8 min, and rinsed thoroughly. Oospores were sown on 0.5% plant agar (P1001; Duchefa Biochemie, Haarlem, the Netherlands) or in glass tubes with *Chara* medium (described previously). The tobacco line BY‐2 (*Nicotiana tabacum* L. cv Bright‐Yellow 2) cells were cultured in a liquid medium containing 3% sucrose, 4.3 g l^−1^ Murashige and Skoog (further referred to as MS) salts (M5524; Sigma‐Aldrich), 100 mg l^−1^ inositol, 1 mg l^−1^ thiamine, 0.2 mg l^−1^ 2,4‐dichlorophenoxyacetic acid, and 200 mg l^−1^ KH_2_PO_4_ (pH 5.8). Cell suspensions were maintained on an orbital shaker (100 rpm) in darkness at 26°C and subcultured weekly. BY‐2 calli stock was grown on the same media solidified with 0.6% plant agar (P1001; Duchefa Biochemie) and subcultured biweekly. *Arabidopsis thaliana* (L.) Heynh. wild‐type (WT) Columbia‐0 (Col‐0) and *pin2* mutant were grown at 22°C with 16 h : 8 h, light : dark cycle on ½MS medium (Murashige & Skoog, 1962). *Marchantia polymorpha* (ssp. *ruderalis*; Causse, 1993) gemmae were grown on 1.4% agar (A111; Phytotech Labs Inc., Lenexa, KS, USA) containing ½B5, at 21°C under constant white light (45 μmol m^−2^ s^−1^ over the waveband 400–750 nm). *Marchantia* gametangiophore stalks were induced by supplementing with continuous far‐red (FR) light (Quantum Devices Inc., Barneveld, WI, USA; 20–40 μmol m^−2^ s^−1^ over the waveband 715–745 nm).

### Chemical treatments

For regeneration experiments, decapitated thallus segments were sown to soil‐sand medium (as described previously) and treated with 1 μM IAA, 10 μM NPA, and the equivalent amount of solvent dimethyl sulfoxide (DMSO) as control. Treatments were repeatedly added to *Chara* medium every 48 h, until the harvest. For metabolic profiling, the treatments were performed on 3‐wk‐old *C. braunii* cultures. Individual plants were treated with 1 μM IAA, 10 μM NPA, and the equivalent amount of solvent (DMSO). The samples were collected after 1, 6, and 24 h.

For cytoplasmic streaming analysis, 3‐wk‐old *C. braunii* cultures were used. The thallus tip containing a whorl of branchlets was cut using scissors and left in its medium for 15 min to recover the streaming upon initial stress from cutting. The thallus tip was then placed on microscopic glass with a drop of *Chara* medium containing 0.1 μM benzoic acid (BA), 1 μM BA, 0.1 μM IAA, 1 μM IAA, and two corresponding concentrations of DMSO as control. The video of cytoplasmic streaming started 1 min after the treatment.

### *In vivo* staining

The PM of internodal and antheridial *Chara* cells was visualized by staining with FM 1–43 dye (stock solution 20 mM in DMSO; Invitrogen) at the final concentration of 5 μM in PBS (140 mM NaCl, 2.95 mM KCl, 2.38 mM KH_2_PO_4_, 7.61 mM Na_2_HPO_4_, pH 6.9) for 5 min and washed 2× with PBS. The PM of BY‐2 cells and *Arabidopsis* was visualized using FM 4–64 (stock solution 20 mM in DMSO; Invitrogen) at the final concentration of 2 μM in its own medium for BY‐2 cells and in water for *Arabidopsis* for 5 min and washed 2× before imaging.

### pH banding

For pH banding, the banding solution (BS) was freshly prepared by adding 0.5 mM NaHCO_3_ to artificial pond water (containing 0.1 mM KCl, 0.1 mM CaCl_2_, and 0.1 mM NaCl; adjusted to pH 6.0) (Zhang *et al*., 2016). The *Chara* culture medium was replaced with the BS, and thalli were allowed to acclimate for 24 h. The following day, the *Chara* thalli were removed from their flasks and transferred to a Petri dish containing BS supplemented with 35 μM phenol red (pH adjusted to 6.0 to achieve a lighter color). The Petri dish was placed on a windowsill to initiate banding. The banding pattern was mapped by photographing, and thalli were subjected to immunostaining. Phenol red appears yellow at pH levels below 6.8 and magenta at pH levels above 8.2.

### Nucleic acid isolation and reverse transcription quantitative polymerase chain reaction

About 50 mg of *C. braunii* thallus, strain S276, was harvested for DNA extraction. The DNA was extracted using the Qiagen DNeasy Plant Mini Kit (for full list of chemicals, see Table S1), and concentration was measured with NanoDrop (Thermo Fisher Scientific, Waltham, MA, USA). RNA was isolated using the FavorPrep Plant Total RNA Mini kit (Favorgen Biotech Corp., Ping Tung, Taiwan) according to the manufacturer's guidelines. DNase treatment was carried out using the DNase set (Macherey‐Nagel, Dueren, Germany). For reverse transcription quantitative polymerase chain reaction (RT‐qPCR), *c*. 1 μg of DNase‐treated RNA was reverse‐transcribed using M‐MLV reverse transcriptase, RNase H‐, point mutant (Promega). Quantitative real‐time PCR was performed using GoTaq qPCR Master Mix (Promega) at 58°C annealing temperature on a LightCycler480 instrument (Roche). PCR efficiency was estimated using serial dilution of template cDNA. *Chara* homolog of *Elongation factor 1a* (Genbank Acc. No. AB607260.1) was used as a reference gene. Positive transcript levels and the quality of PCR products were verified by melting curve analysis.

### Vector construction

The *C. braunii PINa* coding sequence was amplified via PCR from genomic DNA (primers listed in Table S2). As the originally annotated sequence was truncated at the N‐terminus, a forward primer was designed 327 nucleotides upstream. For *CbPINc*, the first exon was amplified, and the rest was synthesized (Biocat, Heidelberg, Germany). Partial sequences were combined by PCR to create a full *CbPINc* sequence. GFP gene (no stop codon) was inserted at the 429^th^ amino acid for CbPINa and 558^th^ for CbPINc. Constructs were cloned into pJET1.2, transformed into *E. coli* (strain JM109), and subcloned into β‐estradiol‐inducible vector pER8‐XVE. To generate plasmids for genetic complementation in *Arabidopsis*, *CbPINa:GFP*, *CbPINc:GFP* fusions, and 1.4 kb PIN2 promoter were separately cloned into the Gateway entry vector pDONR221 and the pPONRP4P1r vector. Constructs were later fused and cloned into Gateway destination vector pB7m24GW.3 by LR reaction. For genetic complementation in *Marchantia*, the cauliflower mosaic virus (CaMV) promoter *pro35S* was cloned into HindIII/XbaI‐cut pMpGWB401 (Ishizaki *et al*., 2015) as described in Fisher *et al*. (2023). This destination vector (pro35S:GW GWB401) was recombined with *CbPINa:GFP* pDonor221 using LR clonase II (Invitrogen) to produce *35S:CbPINa:GFP* GWB401. *LtI6b:eGFP* was amplified from a Level 1 plasmid containing *LtI6b:eGFP* as a DNA template (Pollak *et al*., 2019). The fragment was subcloned into EcoRI‐cut pENTR2b plasmid using a NEBuilderHiFi Gibson Assembly Kit (New England Biolabs, Ipswich, MA, USA). The Gateway destination vector pMpGWB403 (Ishizaki *et al*., 2015) was recombined with pENTR2b using LR clonase II (Invitrogen) to produce *proEF1::LtI6b:eGFP* GWB403.

### Genetic transformation

BY‐2 cells were transformed by cocultivation with *Agrobacterium tumefaciens*, according to the basic transformation protocol (An, 1985). Vectors carrying *XVE::CbPINa:GFP* or *XVE::CbPINc:GFP* were introduced into WT BY‐2 and cultured in MS medium with 100 μg ml^−1^ cefotaxime and hygromycin for selection (Petrášek *et al*., 2003). Transgene expression for microscopy and accumulation assays was induced by adding 2 μM β‐estradiol during inoculation. Transgenic *Arabidopsis* plants were generated using the floral dip method and selected on solid, ½MS medium containing 15 mg ml^−1^ of Basta (Glufosinate). Sporophytes of *M. polymorpha* were collected from a laboratory cross between Mel‐1 and Mel‐2 plants, and male and female WT lines from a Melbourne population, Australia (Flores‐Sandoval *et al*., 2015). For Mp*pin1* sporeling transformations, sporophytes were collected from a laboratory cross between Mp*pin1‐4* and Mp*pin1‐2* plants, as described previously (Fisher *et al*., 2023). Transformation of *Marchantia* sporelings followed the description by Ishizaki *et al*. (2008) using sporeling liquid media ½B5 (G398; Phytotech Labs Inc.) (Gamborg *et al*., 1968).

### Immunohistochemistry

Immunolocalization of *Chara* PINs and H^+^‐ATPase in internodal cells was performed according to Schmölzer *et al*. (2011); fixed internodal cells of thalli were scalpel‐dissected into 3 mm fragments, separated into acidic and alkaline segments; overnight incubated at 4°C with rabbit anti‐H^+^‐ATPase (1 : 1000, AS07260; Agrisera, Vännäs, Sweden), polyclonal rat anti‐CbPINa or anti‐CbPINc (1 : 500; Moravian Biotechnology, Brno, Czech Republic) followed by 2‐h incubation with goat anti‐Rat IgG (H&L; Alexa Fluor 488; A‐11006; Invitrogen) and goat anti‐rabbit IgG (H&L; Alexa Fluor 546; A‐11035; Invitrogen). Antheridia were immunostained according to Żabka *et al*. (2016) using the previously listed antibodies and final staining with DAPI. Microtubules were stabilized with taxol and stained with monoclonal with antibodies against α‐tubulin (DM1A) (1 : 1000) according to Wasteneys *et al*. (1989). Detailed protocols are described in Methods S2–S5.

### Protein extraction and western blot

The procedure described in Schmölzer *et al*. (2011) was followed using rat anti‐CbPINa or anti‐CbPINc (1 : 1000), rabbit anti‐AHA (1 : 2000) and mouse anti‐α‐tubulin as positive controls, rat pre‐immune serum (1 : 1000) as a negative control, and respective secondary HRP‐conjugated antibodies rabbit anti‐rat HRP conjugate (1 : 5000; ENZO ADI‐SAB‐200‐J), goat anti‐rabbit HRP conjugate (1 : 5000; ENZO ADI‐SAB‐300‐J). Proteins were visualized using the enhanced chemiluminescence (ECL) method (Pierce Western Blotting Substrate) and Azure 600 Imaging System. Detailed protocol is described in Methods S6.

### Protein extraction for phosphoproteomic analysis and phosphopeptide enrichment

The protocol was adapted from Kuhn *et al*. (2024). In brief, samples were suspended in extraction buffer containing 100 mM Tris–HCl (pH 8.0), 7 M Urea, 1% Triton‐X, 10 mM DTT, DNase I, MgCl₂, and benzonase. Lysis was performed by sonication, and lysate was cleared by centrifugation. The supernatant was treated with additional benzonase for 30 min at room temperature, followed by alkylation with 50 mM acrylamide for 30 min. Proteins were precipitated using methanol/chloroform (4 : 1 : 3 ratio) with vortexing, followed by centrifugation (5000 ***g***, 10 min). The protein layer was washed with methanol, air‐dried, resuspended in 50 mM ammonium bicarbonate, and quantified using the Bradford assay. For each replicate, 500 μg of protein was digested overnight with trypsin (1 : 100 ratio). Peptides were desalted and concentrated using homemade C18 microcolumns. Phosphopeptides were enriched using Fe‐NTA magnetic beads following the manufacturer's instructions. Peptides were eluted, vacuum‐concentrated, and resuspended in formic acid for analysis. Detailed protocols are described in Methods S7–S9.

### Mass spectrometry

LC‐MS data with all MS/MS spectra were analyzed using the following settings: peptide and protein *FDR* ≤ 0.01; as protein database the proteome of *C. braunii* (UniProt ID UP000265515) was used; variable modifications Oxidation (M), Acetyl (protein N‐term), Deamidation (NQ), pPhospho (STY); fixed modification AcrylAmide (C); maximum missed cleavage was set at 2; match between runs and label‐free quantification options was selected. The maxquant quantitative proteomics software package (Tyanova *et al*., 2016a) was used to analyze LC‐MS data with all MS/MS spectra. Perseus was used for further analysis of the MaxQuant output PhosphoSTY tab (Tyanova *et al*., 2016b).

### Determination of IAA levels and metabolites using LC‐MS

Between 10 and 30 mg of treated *C. braunii* thalli (treatment described previously) was harvested and immediately frozen in liquid nitrogen. The samples were prepared according to Schmidt *et al*. (2024). Hormone analysis was performed with an LC‐MS system consisting of UHPLC 1290 Infinity II (Agilent, Santa Clara, CA, USA) coupled to 6495 Triple Quadrupole Mass Spectrometer (Agilent).

### Radio‐labeled auxin accumulation assay

For accumulation assays, 5‐d‐old estradiol‐induced and noninduced *XVE::CbPINa:GFP* or *XVE::CbPINc:GFP* tobacco BY‐2 cell suspensions were prepared by filtering the liquid phase and resuspending the cells in uptake buffer (Petrášek *et al*., 2003). Cells were incubated in the dark for 45 min, resuspended in fresh buffer to a final concentration of *c*. 700 cells μl^−1^, and incubated for another 90 min. The assay began with the addition of a 2 nM radio‐labeled tracer (NAA), followed by 10 μM NPA, with samples taken every 90 s. Cells were then treated with 500 μl of 96% ethanol for 30 min and mixed with scintillation cocktail (EcoLite(+)™; MP Biomedicals, Irvine, CA, USA). The radioactivity was measured using Tri‐Carb 2900TR scintillation counter (PerkinElmer, Shelton, CT, USA). The NAA accumulation was measured from measured radioactive decay, measured suspension density, and the molar radioactivity of the labeled NAA.

### Gravitropic bending assay

Seedlings were grown on vertical plates under cycles of 16 h : 8 h, light : dark for 4 d. The plates were then turned by 90° and incubated vertically for 1 d. Images were taken after 24 h of gravistimulation, and directional angle of root tips was measured in the imagej software.

### Confocal and brightfield microscopy

*Chara braunii* brightfield images of thallus and antheridia were acquired on Olympus Provis AX‐70 (Olympus, Tokyo, Japan) with a color digital camera (DP28; Olympus) using the DP2‐AOU imaging software. Confocal laser scanning microscopy was performed using Leica SP8 (Leica, Wetzlar, Germany) with the HC PL APO CS2 ×63/1.2 W objective (GFP, Alexa Fluor 488, FM 1–43: λ_ex_ = 488 nm, λ_em_ = 490–550; Alexa Fluor 546: λ_ex_ = 546, λ_em_ = 570–630; FM 4–64: λ_ex_ = 488 nm, λ_em_ = > 590 nm, DAPI, calcoflur and Hoechst 33342 λ_ex_ = 405, λ_em_ = 390–490 nm), the pinhole was set to 1 AU at 580 nm. The equatorial sections of estradiol‐induced 5‐d‐old BY‐2 cells expressing XVE:CbPINa:GFP or XVE:CbPINc:GFP were acquired by Leica SP8 confocal microscope (HC PL APO CS2 ×63/1.2 W objective, image resolution *c*. 5 px μm^−1^) with the same detection range as for *C. braunii*. The confocal images of *A. thaliana* were detected using confocal microscope (LSM 800, inverted; Zeiss). For imaging GFP and FM 4–64, the 488 nm laser (GFP: λ_em_ = 500–550, FM 4–64: λ_em_ = 670–740 nm) was used. The confocal images of *M. polymorpha* were taken with a Leica SP5 upright confocal microscope (GFP: λ_ex_ = 488 nm, λ_em_ = 505–515). The objective used was a ×10 NA 0.30 dipping lens (HCX APO L U‐V‐I 10×/0.30); the agar plate growing 1‐d‐old gemmalings was filled with water immediately before imaging. Gametangiophore stalks were cut down the center of the stalk with a razor blade before mounting to a plate lid with Blu‐Tack which was filled with water for imaging. For white light images of *Marchantia* gametangiophore stalks, a stereolumar v.12 dissecting microscope, 90.63 FWD 81 mm PlanApo S objective with camera AxioCamHRc (Zeiss), was used. All images were processed using fiji (imagej) by creating z‐stacks, selecting channels colors under ‘Lookup Tables’, and adjusting ‘Brightness/Contrast’ settings.

### Quantification of plasma membrane fluorescent signal

PM affinity of PIN proteins in BY‐2 cells expressing CbPINa:GFP and CbPINc:GFP was quantified by a single‐dimensional deconvolution‐based method based on comparison of distribution of GFP‐labeled PIN, cytoplasmic GFP, and FM 4–64 signals along the profiles taken across PM (Vosolsobě *et al*., 2017).

### *In silico* molecular docking

Protein structures were predicted using colabfold (Mirdita *et al*., 2022), which integrates multisequence alignment with alphafold (Jumper *et al*., 2021). The structures were relaxed in water using the CHARMM36 force field (Best *et al*., 2012) and GROMACS (Abraham *et al*., 2015) and visualized with ChimeraX (Goddard *et al*., 2018). Ligands were docked using AutoDockFR (Zhao *et al*., 2006; Ravindranath *et al*., 2015), initially placed based on IAA or NPA poses in AtPIN8 (Ung *et al*., 2022). Affinity grids were automatically set with 4.0 Å padding and 0.375 Å spacing. Each docking included 50 runs with 25 million evaluations, and results were visualized using LigPlot+ (Laskowski & Swindells, 2011).

### Multiple sequence alignment

The *Arabidopsis* PIN1 (AT1G73590), *Marchantia* PIN1 (Mapoly0089s0050), and *C. braunii* PINa and c were aligned in Geneious Prime 2021.2.2 using muscle 3.8.425 (Edgar, 2004) algorithm.

### RNA‐seq quantification

Publicly available transcriptomic libraries (a list is available in the GitHub repository) were quantified by two independent approaches: first, Kallisto 0.48.0 with *C. braunii* CDS from genome GCA_003427395 as a reference was used; and second, reads were aligned to the genomic reference GCA_003427395 by 2‐pass mapping in STAR 2.7.10 and next quantified in RSEM 1.3.3 with option for strand‐specific protocol, if the ratio of reads mapper to sense and antisense strain was > 10 in STAR quantification. Only the genes with *TPM* > 0.5 were considered as expressed.

## Results

### IAA induces more axillary branches in nodes of decapitated *Chara* thalli

**Fig. 1:**
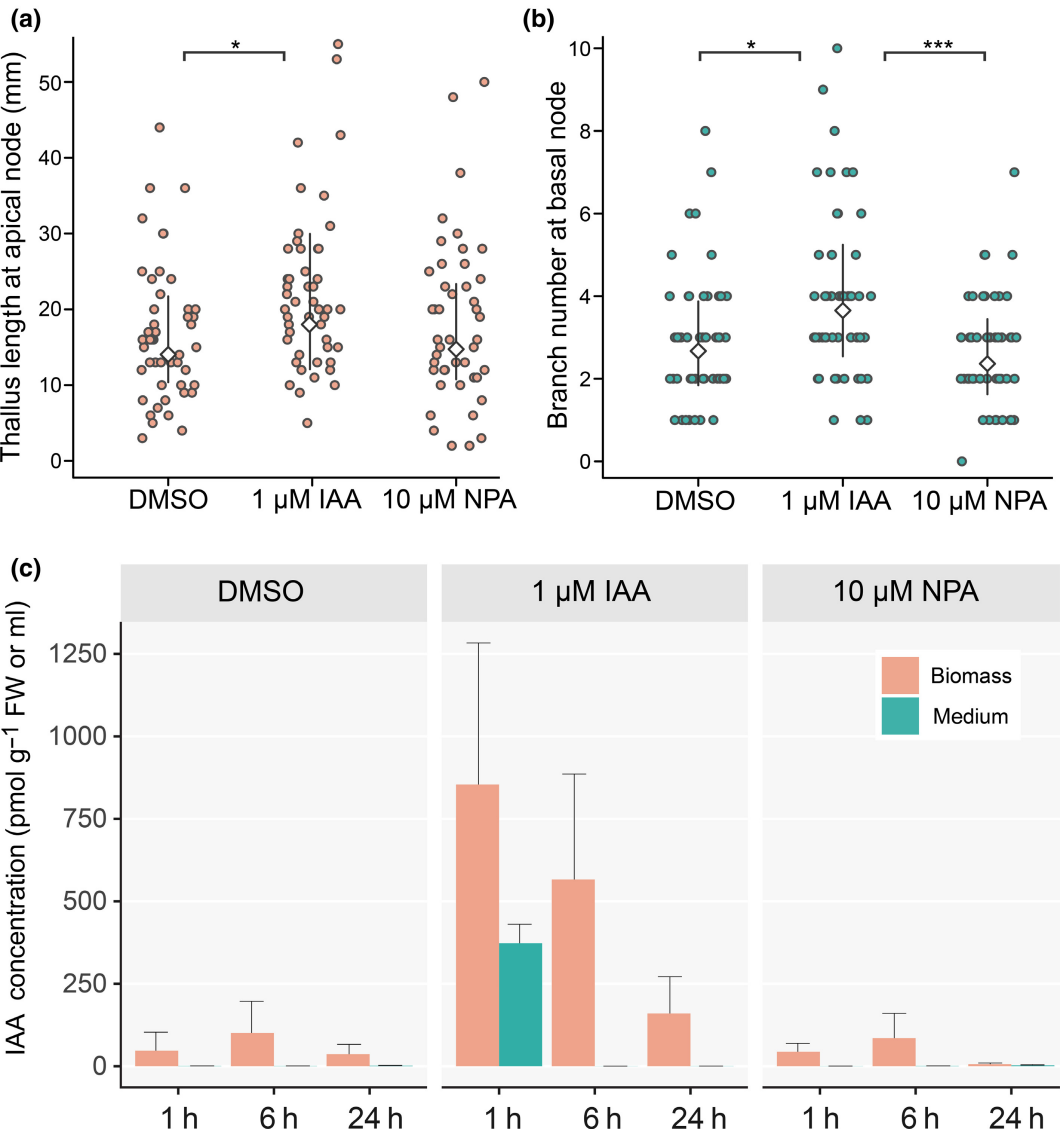
Decapitated *Chara braunii* explants react to indole‐3‐acetic acid (IAA) and metabolize it quickly. (a) Newly regenerated thallus length following treatments with dimethyl sulfoxide (DMSO), 1 μM IAA, 10 μM naphthylphthalamic acid (NPA). The length of thallus was measured from the apical nodal complex of an explant to the regenerated thallus tip 15 d after sowing the explants. (b) Number of axillary branches at the basal node upon treatments with DMSO, 1 μM IAA, 10 μM NPA. The number of the side branches was counted under microscope 15 d after sowing the explants. The effect of the treatments for (a, b) were analyzed by a generalized linear mixed effects model and the results of pairwise comparisons are shown (*, *P* < 0.05; ***, *P* < 0.001) with means and their 95% confidence intervals indicated in the plots. (c) LC‐MS determined concentration of IAA in *Chara* biomass and media collected after 1, 6, and 24 h of treatments with DMSO, 1 μM IAA and 10 μM NPA. Error bars show ±SD.

Although IAA effects on rhizoid growth promotion (Klämbt *et al*., 1992) and antheridia mitosis (Godlewski, 1980) have been shown in previous studies, there has not been an attempt to identify the morphogenic role of IAA. Considering NPA‐sensitive auxin transport has been previously shown in *Chara* (Boot *et al*., 2012), we tested whether external applications of IAA or NPA cause morphological changes. We focused on the process of regeneration of new thallus upon decapitation, which is derived from nodal cells whose cell initials possess stem cell‐like capacity (Heß *et al*., 2023b). *Chara* regenerated longer thallus (Fig. 1a) when decapitated explants containing two nodal complexes connected with one internodal cell (Fig. S2a) were treated with 1 μM IAA, while treatments with NPA had no effect. Furthermore, we tested whether IAA is indeed ineffective in restoring the apical dominance after the tip decapitation, as shown previously (Clabeaux, 2011). After decapitation, we counted the number of axillary branches that developed from nodal cells at the base of thallus and found, surprisingly, that there were significantly more branches present after IAA application (Fig. 1b), and NPA again had no effect (Fig. 1b).

As we have shown recently (Schmidt *et al*., 2024), there is evidence for biosynthesis and metabolism of IAA in *C. braunii*. We were therefore interested in the fate of applied IAA and noted that it was completely depleted from the medium only 6 h after treatment (Fig. 1c). By contrast, IAA highly accumulated in *Chara* biomass, where it decreased during a 24‐h incubation to levels of DMSO‐treated controls (Fig. 1c). We also observed the formation of IAA metabolites (Fig. S3a,b). Interestingly, under NPA treatment, IAA levels in the biomass after 24 h were almost depleted (Figs 1c, S3c). Altogether, these results suggest that exogenous IAA is taken up by *Chara*, and it has morphogenic effects.

### Identification of six *CbPIN* paralogs and their expression

**Fig. 2:**
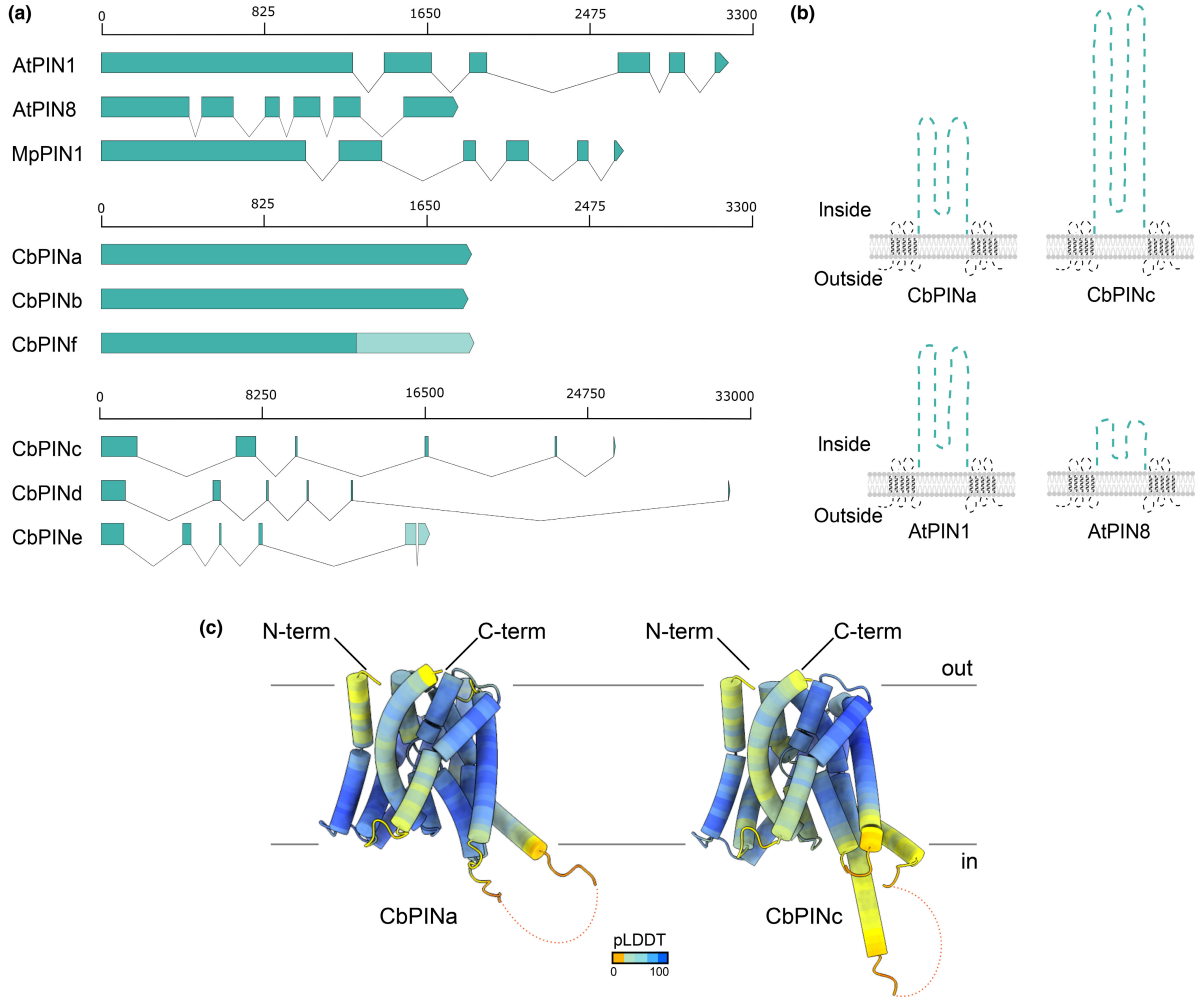
*Chara braunii* has six PIN homologs. (a) Exon–intron gene structures of *Arabidopsis thaliana PIN1 and PIN8*, *Marchantia polymorpha PIN1* and six *C. braunii PINs*, intron‐less *CbPINa*, *b*, and *f*, and long intron‐containing *CbPINc*, *d*, and *e*. Light green represents the missing regions or uncertain regions of the gene. (b) Schematic representation of secondary structures of CbPINa and CbPINc in comparison with the *Arabidopsis* AtPIN1 and AtPIN8. The predictions were performed using DeepTMHMM. (c) Predicted 3D structures of CbPINa and CbPINc proteins using alphafold. Color coding by pLDDT represents the confidence of the predicted structure compared to the true structure. Hydrophilic loop is unstructured and removed.

The effects of IAA on branching and NPA‐specific changes in IAA suggested the presence of specific auxin transporters, presumably involved in the auxin flow in *Chara*. Following the approach used in the functional analysis of PIN homolog from *Klebsormidium flaccidum* (Skokan *et al*., 2019), we aimed to clone PIN genes from *C. braunii*. The whole genome sequencing revealed that *C. braunii* has six PIN homologs: g29962, g29961, g41200, g50423, g50425, and g84230 (Nishiyama *et al*., 2018). We named the genes *CbPINa* to *CbPINf* (Table S3). Compared with land plant *PINs*, whose introns are a couple of hundred bases in length, *C. braunii PIN*s are either intron‐less (*PINa*, *PINb*, and *PINf*) or contain long introns (*PINc*, *PINd*, and *PINe*) from several thousand up to almost 20 000 base pairs in length (Fig. 2a). *In silico* predictions of secondary protein structures of these two *Chara* PINs showed that PINa has a long hydrophilic loop corresponding to the size the loop in canonical AtPIN1, while CbPINc has an extremely long cytosolic loop (> 1000 amino acids) (Fig. 2b). Both *Chara* PINa and PINc have a canonical structure of 10 highly conserved transmembrane domains (Fig. 2c).

To analyze the expression patterns of individual *CbPINs*, we screened all publicly available *C. braunii* RNA‐seq data (Fig. S4b), which indicated preferential expression of *CbPINa* in protonema and rhizoids, while *CbPINc* was expressed in all analyzed stages besides rhizoids and oospore (Fig. S4b). We confirmed these results by RT‐qPCR (Fig. S4a) from cDNA isolated from vegetative cells, where only *PINa* and *PINc* were successfully amplified and their identity verified by sequencing. Based on these results, we further focused on the functional characterization of *Chara PINa* and *PINc*.

### *Chara braunii* PINs are localized the acid bands of charasome PM

**Fig. 3:**
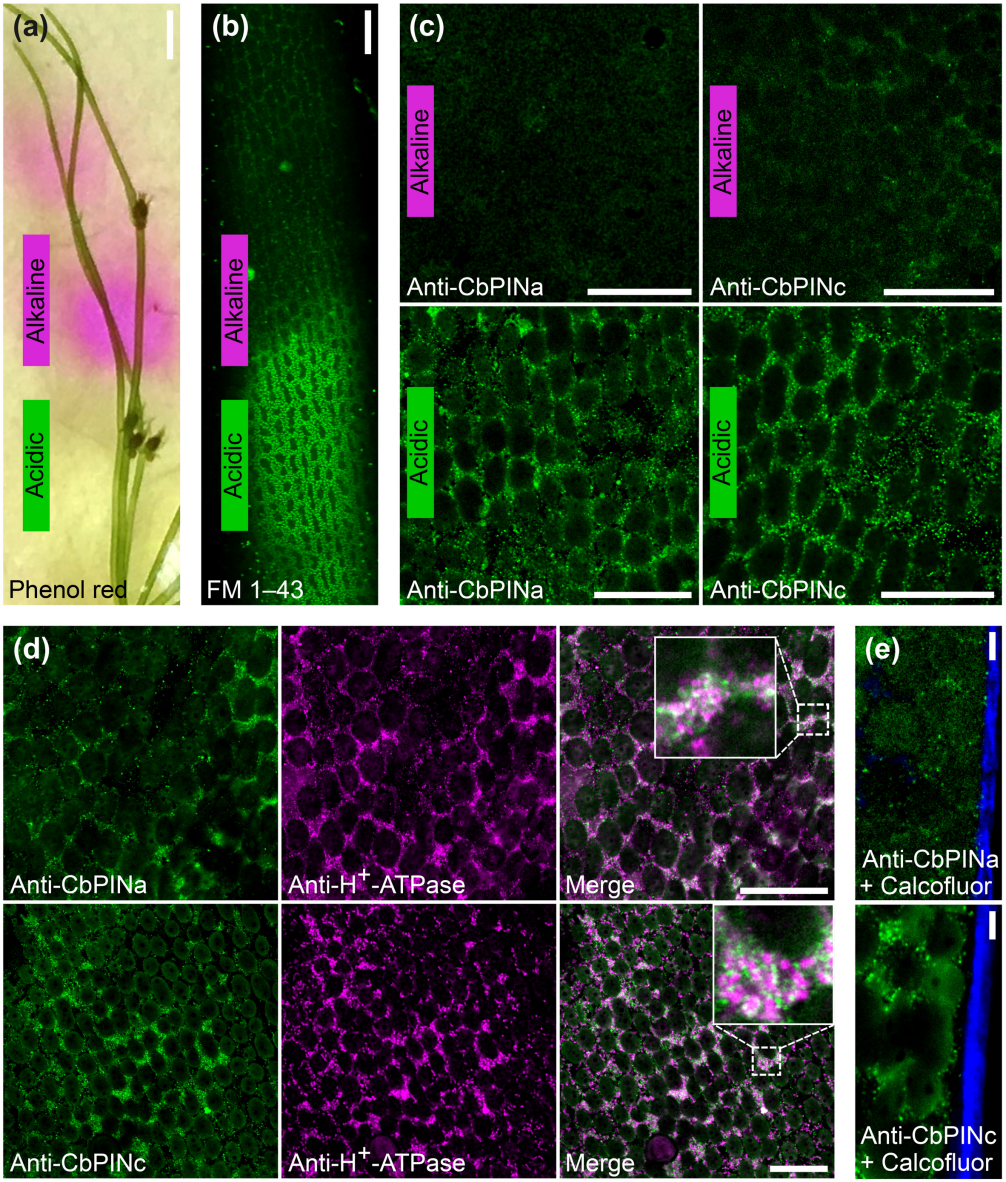
Immunolocalization of CbPINa, CbPINc, and H^+^‐ATPase in the charasome plasma membrane of *Chara braunii* internodal cells. (a) pH banding of *C. braunii* branchlet internodal cells visualized with phenol red. The magenta color indicates alkaline regions with acidic regions in between. (b) *In vivo* staining of *C. braunii* internodal cell with FM 1–43. Note the intensity of FM 1–43 dye in the acidic region indicating the abundance of charasome PM. (c) Indirect immunofluorescence staining of CbPINa and CbPINc in the alkaline and acidic regions of an internodal cell. The fluorescent signal at the cell surface is present in acidic regions forming a charasome pattern and absent in the alkaline region. (d) Indirect immunofluorescence co‐staining of CbPINa and CbPINc (green) with H^+^‐ATPase (magenta). Note the partial colocalization (white) zoomed in. (e) Indirect immunofluorescence staining of CbPINa and CbPINc, co‐stained with calcofluor‐white cell wall dye. Bars: (a) 100 μm; (b–d) 20 μm; (e) 5 μm.

NPA‐sensitive auxin transport independent of cytoplasmic streaming has been demonstrated previously in internodal cells of *C. corallina* (Boot *et al*., 2012). In land plants, this transport occurs via PM‐localized PIN auxin efflux carriers (Ung *et al*., 2023). Therefore, we decided to localize *Chara* PINs in the internodal cells that form charasomes, convoluted PM domains (Chau *et al*., 1994) abundant in acidic regions of internodal cells (Schmölzer *et al*., 2011). As there are currently no reliable protocols for stable genetic transformation of *Chara* (Kurtović *et al*., 2024), we generated epitope‐specific PIN polyclonal antibodies against CbPINa and CbPINc and confirmed their specificity by western blotting (Fig. S5b). To map the acid and alkaline bands, we stained *C. braunii* thalli with the pH‐sensitive dye phenol red (Fig. 3a) and visualized the charasome PM using FM 1–43, which revealed a characteristic clustered PM pattern (Fig. 3b). Charasomes were highly abundant and exhibited strong FM 1–43 fluorescence in the acidic region. By contrast, alkaline regions contained fewer and less fluorescent charasomes (Fig. 3b). This observation is consistent with previous PM staining in *Chara* (Schmölzer *et al*., 2011). Next, we investigated whether *Chara* PINs localize to acidic or alkaline regions. Indirect immunofluorescence staining of both CbPINa and CbPINc in the acidic region of internodal cells followed by confocal microscopy showed specific dotted signals (Fig. 3c), reminiscent of charasomes. In line with the FM 1–43 staining, CbPINa and CbPINc signals were nearly absent in alkaline regions, except for occasional PIN‐containing dots (Fig. 3c). We did not observe any specific signals in the controls, including the omission of primary antibodies or in samples probed with the pre‐immune serum (Fig. S5a). To further confirm the identity of the PM signal, we costained CbPINa and CbPINc with antibodies against H^+^‐ATPase, which was shown previously to reside on charasomes (Schmölzer *et al*., 2011). The fluorescence signals of both CbPINa and CbPINc showed partial colocalization with H^+^‐ATPase (Fig. 3d) in charasomes, indicating that two *Chara* PINs are indeed bound to specific regions of the PM. However, the colocalizations were only partial (see insets in Fig. 3d). Importantly, we did not observe any unspecific binding of antibodies to the cell wall (Fig. 3e). Overall, we show that *Chara* PINa and PINc are preferentially localized within the charasome PM compartments of acidic regions in internodal cells and partially colocalize with H^+^‐ATPase.

### *Chara braunii* PINs are localized in the PM of antheridial cells

**Fig. 4:**
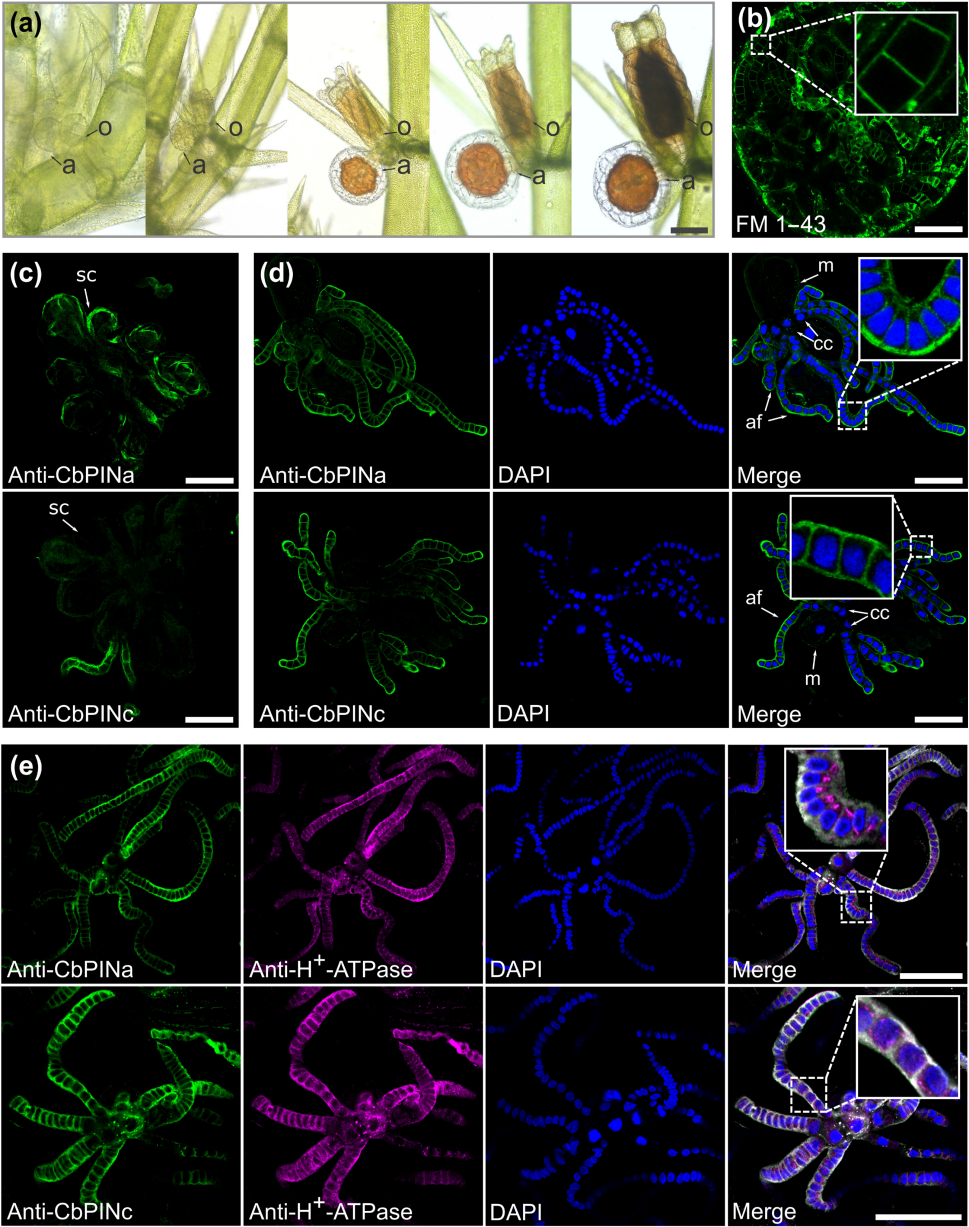
Immunolocalization of CbPINa, CbPINc, and H^+^‐ATPase in the plasma membrane (PM) of antheridial cells of *Chara braunii*. (a) Progression of antheridium development on *C. braunii* thallus. a, antheridium; o, oogonium. (b) *In vivo* staining of antheridium with FM 1–43. Inset shows the magnification of a filament with a smooth PM. (c) Differential presence of CbPINa and CbPINc in shield cells (sc), where CbPINa signal resides on the edges of the cell and there is no CbPINc signal. (d) Antheridial filament stained with either CbPINa or CbPINc (green), DAPI staining of the nucleus (blue) and merged image with inset. af, antheridial filament; cc, capitular cells; m, manubrium. (e) Costaining of CbPINa and CbPINc (green) with H^+^‐ATPase (magenta) and nuclear staining with DAPI (blue). Merged images include all three channels and an inset of antheridial filament cells. Bars: (a) 300 μm; (b) 100 μm; (c–e) 50 μm.

The analysis of *C. braunii PIN* expression from publicly available RNA sequencing profiles showed that, besides thallus, *CbPINa* and *CbPINc* were also expressed in male generative organs, antheridia (Fig. S4). The PIN homolog presence in antheridia was suggested by using heterologous antibodies against *A. thaliana* PIN2 in *C. vulgaris* (Żabka *et al*., 2016). The antheridia consist of generative antheridial filaments and nongenerative shield cells, which encapsulate rosette‐forming filaments (Fig. 4a). Antheridial filaments have a continuous PM that can be stained *in vivo* with FM 1–43 (Fig. 4b). Our immunolocalizations showed that CbPINa, but not CbPINc, was present in shield cells (Fig. 4c). Furthermore, we localized both CbPINs at the edges of antheridial filaments (Fig. 4d), in a nonpolar manner (insets in Fig. 4d). The signal was stronger toward the ends of filaments and was not present in capitular (progenitor cells), nor in manubria. We confirmed the identity of PM signal by costaining with H^+^‐ATPase antibodies, which showed that H^+^‐ATPase does localize to the cell in the same regions as PINs at the edges of the cells, but the signal was also present inside cells, around the DAPI‐stained nucleus (insets in Fig. 4e). We did not observe any specific signals in the controls, including the omission of primary antibodies or the inclusion of pre‐immune serum (Fig. S5c,d), while immunostaining of α‐tubulin served as a positive control (Fig. S6). Overall, the specific localization of CbPINa and CbPINc at the PM and their colocalization with H^+^‐ATPase suggests that these proteins play a role at the PM of male generative cells.

### CbPINa and CbPINc functional assays in tobacco BY‐2 cells

**Fig. 5:**
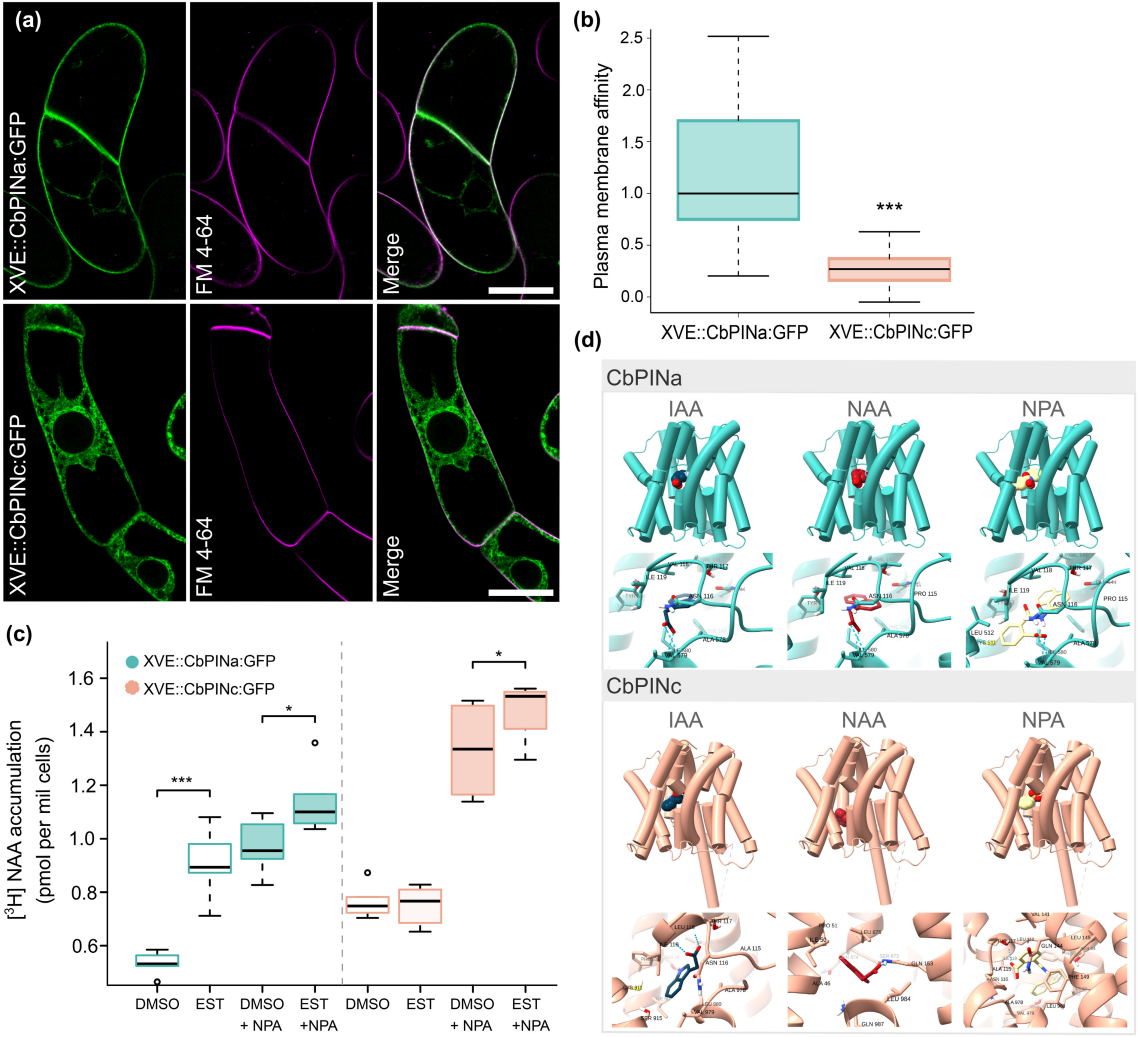
CbPINa localizes preferentially to the plasma membrane of BY‐2 cells and interferes with radio‐labeled 1‐naphthaleneacetic acid ([^3^H]‐NAA) accumulation while endoplasmic reticulum (ER)‐localized CbPINc does not. (a) Induced XVE::CbPINa:GFP and XVE::CbPINc:GFP BY‐2 cells. Shown are following: the GFP signal at the plasma membrane (PM) and ER in XVE::CbPINa:GFP and predominantly ER in XVE::CbPINc:GFP, FM 4–64 PM staining, and a merged image. (b) The comparison of *Chara braunii* PINs PM affinity. Boxplots show median, first and third quartiles, and 95% confidence intervals of medians. *n* = 10 cells per variant (ANOVA on log‐transformed data: ***, *P* < 0.001). (c) [^3^H]‐NAA accumulation in 5‐d‐old induced and noninduced XVE::CbPINa:GFP and XVE::CbPINc:GFP cells after 10 min of accumulation assay. Naphthylphthalamic acid (NPA)‐sensitive NAA efflux was tested with the NPA (10 μM) applied at the beginning of each run of accumulation assay. Boxplots show median, first and third quartiles, and 95% confidence intervals of medians. Dashed line indicates experiments were performed in independent runs. Data from two biological repetitions, with three technical repetitions, were analyzed by linear mixed effects model and results of pairwise comparison is indicated (*, *P* < 0.05; ***, *P* < 0.001). (d) Ligand docking of indole‐3‐acetic acid, NAA, and NPA to *C. braunii* PINa and PINc. 3D models show the position of the ligand within the protein and a close‐up view shows the support sites with central residues highlighted. Predicted intermolecular hydrogen bonds are shown as dashed lines. Bars, 25 μm.

To test the function of CbPINs, we introduced GFP‐tagged *CbPINa* and *CbPINc* coding sequences under the control of the estrogen inducible XVE system (Zuo *et al*., 2000) and expressed them heterologously in tobacco BY‐2 cells. Such XVE::CbPINa:GFP and XVE::CbPINc:GFP cell lines were used for confocal microscopy and auxin transport accumulation assays (Petrášek *et al*., 2006; Müller *et al*., 2019), following the setup that we used previously for testing PIN homolog from *K. flaccidum* (Skokan *et al*., 2019). Following induction, we observed a preferential localization of CbPINa at the PM, where it colocalized with FM 4–64 (Figs 5a, S7a) and a weak signal at the endoplasmic reticulum (ER). By contrast, CbPINc was predominantly localized to the ER, with a significantly lower PM localization compared with CbPINa (Figs 5b, S7a). In the same induced cells, we performed auxin transport assays using radio‐labeled 1‐naphthaleneacetic acid ([^3^H]‐NAA), a lipophilic synthetic auxin with very good specificity for auxin efflux carriers and preferred synthetic auxin for tracing carrier‐mediated auxin efflux (Delbarre *et al*., 1996; Petrášek *et al*., 2006). Surprisingly, the induced BY‐2 line expressing CbPINa showed higher accumulation of [^3^H]‐NAA, almost reaching levels observed after the inhibition of endogenous auxin efflux with NPA (Figs 5c, S7b). This suggests that CbPINa, while inactive as a carrier in tobacco cells, interfered with the activity of endogenous auxin efflux carriers, possibly by occupying their native residence in the PM and binding NAA. By contrast, the induced BY‐2 line expressing CbPINc showed no difference in [^3^H]‐NAA levels (Figs 5c, S7b).

Interestingly, despite CbPINa and CbPINc not showing auxin efflux activity in tobacco cells, multiple sequence alignment with land plant PINs (AtPIN1 and MpPIN1) (Fig. S8) revealed that they both possess a conserved phosphorylation site S2, which is crucial for regulating auxin efflux‐dependent growth (Weller *et al*., 2017). To test the affinity of CbPINs to IAA, NAA, and NPA, we performed an *in silico* docking experiment (Fig. 5d). The ligands were predicted to dock into the expected binding cavity of both CbPINa and CbPINc, located near the crossed transmembrane helices (Su *et al*., 2022; Ung *et al*., 2022). The binding sites for IAA on CbPINs showed interactions with several conserved amino acids involved in auxin transport of *A. thaliana* PINs (Su *et al*., 2022; Ung *et al*., 2022), namely Asn116, Ile580, and Val579 in CbPINa, and Leu118 and Ile119 in CbPINc. Moreover, the predicted binding affinities of IAA, NAA, and NPA for CbPINa and CbPINc were comparable to those of PIN3 from Arabidopsis (Table S4), a PIN efflux carrier with an experimentally resolved structure (Su *et al*., 2022). In conclusion, our results suggest PM‐localized CbPINa interferes with the PM‐based auxin transport machinery, in contrast to predominantly ER‐resident CbPINc.

### *Chara* PIN heterologous expressions in *Arabidopsis* and *Marchantia*

**Fig. 6:**
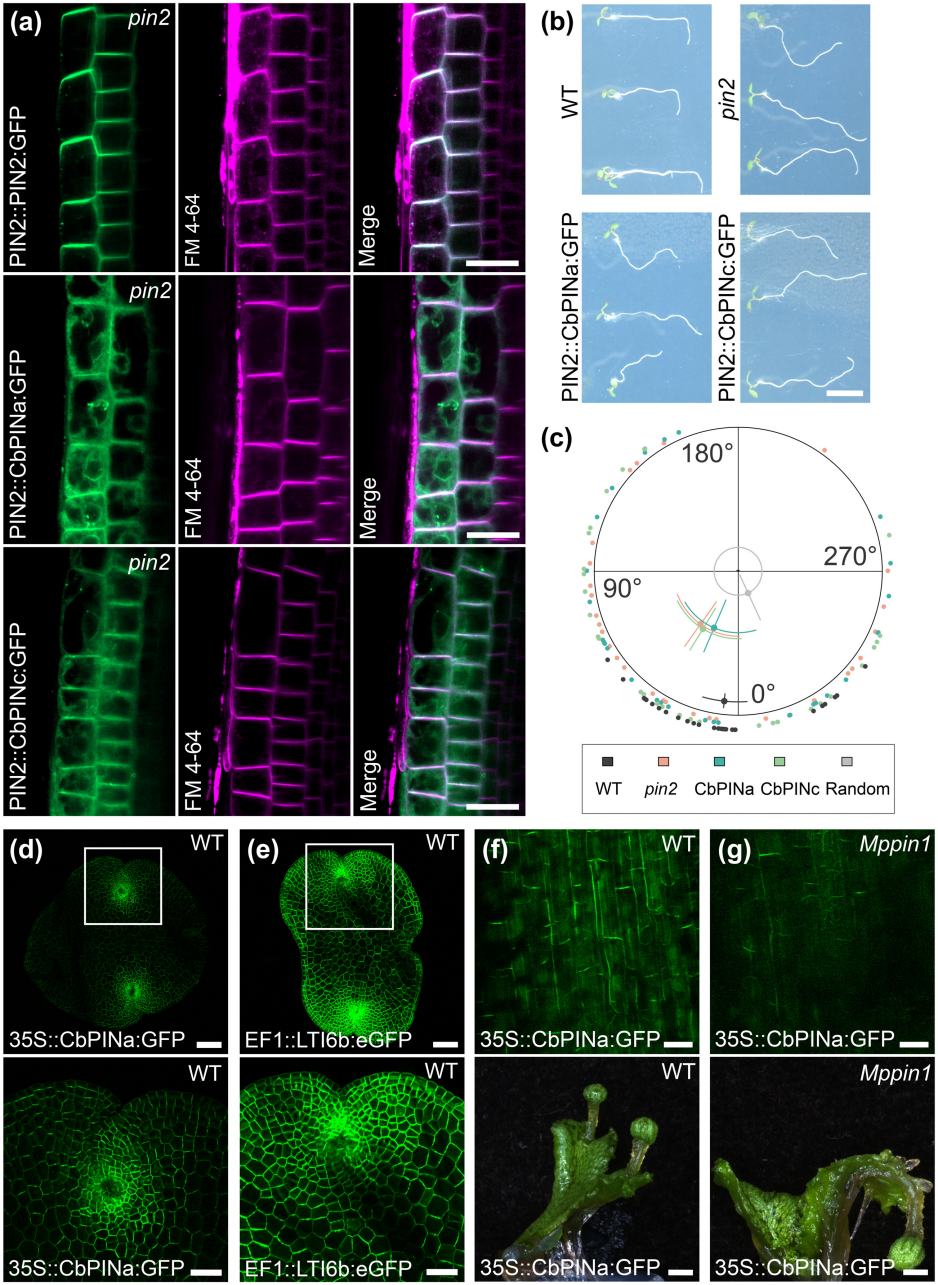
CbPINa and CbPINb do not complement *Arabidopsis thaliana* and *Marchantia polymorpha* mutant phenotypes. (a) Localization of PIN2::PIN2:GFP/*pin2*, PIN2:CbPINa:GFP/*pin2* and PIN2:CbPINc:GFP/*pin2* in *A. thaliana* root epidermal cells. Nonpolar localization of CbPINa and CbPINa in the plasma membrane (PM) and endoplasmic reticulum (ER), shootward AtPIN2 localization (b) Gravitropic root bending in 4‐d‐old seedlings of the wild‐type (WT), *pin2* mutant, and PIN2::CbPIN‐expressing lines. (c) Quantification of the root bending assay. Root‐tip angles (*n* = 30 per variant) are plotted in the outer section of the circular diagram, while the inner section displays points representing the mean angle and the length of the directional vector. The directional vector length reflects the dominance of a given direction on a 0–1 scale, as defined by the circle's diameter. Confidence intervals (*CIs*) are shown for both the mean angle (as an arc segment) and the directional vector length (as a straight line). CIs were estimated through bootstrapping (*n* = 1000 iterations of the original data). The variant labeled ‘random’ illustrates confidence intervals derived from 1000 sets of randomly generated angles (*n* = 30) for comparison with the root angle distributions in the analyzed variants. (d) *Marchantia polymorpha* gemmaling (upper) and zoomed‐in gemmaling notch (lower) expressing 35S::CbPINa:GFP/WT. The GFP signal is predominantly present on PM. (e) EF1::LTI6b:GFP/WT as a PM marker. Note the uniform localization across gemmaling. (f) Close‐up image of a female gametangiophore stalk expressing 35S::CbPINa:GFP/WT (upper) and white image of its corresponding thalli with female gametangiophore (lower). (g) Close‐up image of a female gametangiophore stalk expressing 35S::CbPINa:GFP/Mp*pin1* (upper) and white light image of its corresponding thalli with female gametangiophore (lower). Note the agravitropic stalk in the Mp*pin1* mutant background. Bars: (a) 20 μm; (b) 500 μm; (d, e) 100 μm (upper) and 50 μm (lower); (f, g) 50 (upper) and 2 mm (lower).

Although the functional PIN auxin exporter from *K. flaccidum* (Skokan *et al*., 2019) does not complement gravitropism defects when expressed under PIN2 promoter in *Arabidopsis* roots (Zhang *et al*., 2019), based on our results from tobacco cells, we decided to test the possible role of CbPINa and CbPINc in Arabidopsis. We first generated *Arabidopsis pin2* mutant plants expressing *PIN2::CbPINa:GFP* and *PIN2::CbPINc:GFP* (Fig. 6a). Using these lines, the nonpolar localizations of both CbPINa:GFP and CbPINc:GFP in the PM and ER were observed in the root epidermal cells, while PIN2:GFP showed a known apical (shootward) polar PM localization (Fig. 6a). These localizations corresponded to the results of gravitropic bending experiments, where CbPINa:GFP and CbPINc:GFP did not rescue the agravitropic phenotype of *pin2* mutant (Fig. 6b,c).

In order to test the role of *Chara* PINs in a less complex organism, we expressed CbPINa:GFP under the control of 35S promoter in the bryophyte model *M. polymorpha*, which contains just one canonical, long hydrophilic loop PIN (MpPIN1) (Fisher *et al*., 2023). Interestingly, CbPINa localized predominantly to the PM in *M*. gemmaling (Fig. 6d), and its distribution was, in comparison with the LTI6b PM marker (Fig. 6e), more concentrated on the gemmaling notches. Surprisingly, female gametangiophore stalk CbPINa localized to the PM in a polar manner (Fig. 6f); nonetheless, it did not complement the Mp*pin1* mutant phenotype (Fig. 6g). Altogether, although *Chara* PINs could be localized at the PM in *Arabidopsis* and *Marchantia*, they do not complement the functions of canonical land plant PINs.

### IAA enhances cytoplasmic streaming and triggers global phosphorylation response in *C. braunii*

**Fig. 7:**
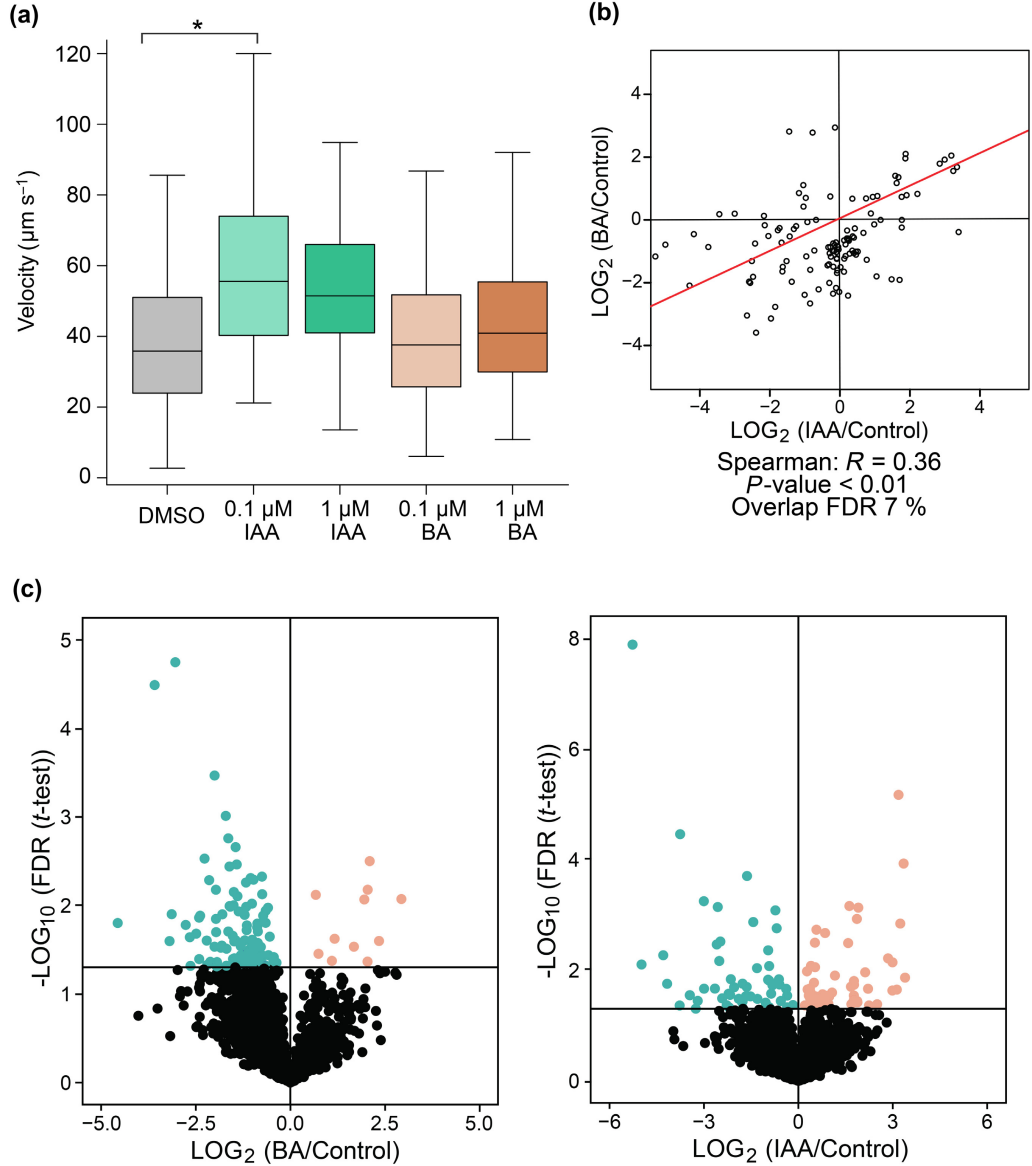
Indole‐3‐acetic acid (IAA) enhances *Chara braunii* cytoplasmic streaming and induces a specific phosphoproteomic response. (a) Boxplot of manually tracked velocities in branchlets of 3‐wk‐old thallus tips of *C. braunii*. Data representing 10 s were acquired 1 min after the treatment and analyzed by linear mixed effects model. Boxplots show median, first and third quartiles, and 95% confidence intervals of medians (*, *P =* 0.02318). The asterisk‐marked variants have the most dominant pairwise difference determined by estimated marginal means (*P* = 0.0840) and linear mixed effects model, when analyzed separately (*, *P* = 0.03896). (b) Plot comparing the differential phosphosites (*FDR* ≤ 0.05) after a 2‐min treatment with 0.1 μM IAA (*x*‐axis) to the fold‐change of the corresponding phosphosites following a 2‐min treatment with 0.1 μM benzoic acid (BA). The red line represents the regression line. (c) Distribution plots of significant differential phosphosites comparing 2 min of 0.1 μM IAA treatment with dimethyl sulfoxide (DMSO) treatment and 0.1 μM BA treatment with DMSO treatment. Orange color represents hyperphosphorylated phosphosites, blue represents hypophosphorylated, while black represents detected but not significant sites.

Recent work has demonstrated that low concentrations of IAA trigger a specific and fast phosphoproteomic response in both streptophyte algae and land plants within 2 min of treatment. This response correlated specifically with the effect of low concentration of IAA on cytoplasmic streaming (Friml *et al*., 2022; Kuhn *et al*., 2024). Therefore, we performed a set of cytoplasmic streaming observations on branchlet internodal cells in 3‐wk‐old *Chara* thalli. (Fig. 7a). Branchlets are smaller than the internodal cells of main axis and more suitable for microscopic observations. The cytoplasmic streaming velocity increased significantly in cells treated with 0.1 μM IAA as compared to DMSO treatment, while 1 μM IAA, 0.1 μM BA, and 1 μM BA caused no significant change in streaming velocity (Videos S1– S5). To obtain the most robust results, we applied a highly conservative approach using a linear mixed effects model, which considered all random effects (individual cells and replications) and minimized false‐positive results. In this case, we got only a slightly significant difference (*, *P* = 0.02318) compared with standard one‐way ANOVA (***, *P* < 2.2e−16) and nonsignificant pairwise comparison (EMMs) with the highest difference being between treatments with 0.1 μM IAA vs DMSO. Building on this observation, we conducted a phosphoproteomic analysis to understand if these fast IAA‐triggered changes in cytoplasmic streaming are accompanied by similar IAA‐specific phosphorylation modifications of proteins as has been shown previously in *Klebsormidium* and *Penium* (Kuhn *et al*., 2024). Furthermore, we used BA as control for the general effect of treatment by aromatic acid. Our results revealed that 0.1 μM IAA, after just 2 min of treatment, induces specific phosphorylation changes, as shown by a weak correlation with BA treatment (Fig. 7b). Furthermore, IAA induced specific shifts in both phosphorylation and dephosphorylation states of *Chara* proteome (Fig. 7c). While the number of detected sites was almost 2000, the numbers of differential phosphosites were 114 in IAA vs DMSO (Tables S5, S6), comparison at false discovery rate *FDR* ≥ 0.05. Furthermore, hyperphosphorylation after IAA treatment represented the majority of differential phosphosites (55.10%), while after BA, only 9.48% differential phosphosites were hyperphosphorylated. Although *Chara* PINs were not detected in our dataset, we identified a distinct set of candidates potentially involved in auxin response. The most strongly influenced candidate is homolog of MAP4K class III (A0A388LCC7), dephosphorylated at Ser793 and Ser332. Notably, our data indicate that the *Chara* homolog of a RAF‐like kinase (A0A388MAZ7) was phosphorylated by auxin at Ser1509, further confirming its deeply conserved evolutionary role in rapid auxin responses. To elucidate how the differentially phosphorylated candidates are involved in known and predicted protein–protein interactions, we performed a STRING analysis (Fig. S9), which revealed clusters of proteins involved in autophagy, transcriptional regulation, endocytosis, and cation transport, namely sodium/hydrogen exchanger (A0A388MCN6; hyperphosphorylated at Ser499). Together, these findings highlight a rapid and highly specific auxin‐triggered phosphorylation response in *Chara*, pointing to conserved mechanisms in auxin signaling across streptophyte lineage.

## Discussion

### The morphogenic response of *C. braunii* to IAA

Studying the evolutionary origins of auxin biology and responses is one of the most fundamental tasks of plant evo‐devo (Bowman *et al*., 2021). Auxin, primarily IAA, is known to play a crucial role in regulating growth and development in land plants, particularly in processes such as cell elongation, organogenesis, and apical dominance (Friml, 2022). Understanding how auxin responses originated and evolved would shed light on the evolution of plants' morphological complexity. In this context, our study explores the IAA role in *C. braunii*, a late‐diverging streptophyte alga, which has emerged as a model system for studying plant evolution (Holzhausen *et al*., 2022). Previous research in *Chara* showed that IAA promotes rhizoid growth (Klämbt *et al*., 1992) and that there is branching induction after thallus tip decapitation in *Chara*, reflecting early forms of apical dominance (Clabeaux, 2011). In this study, we tested the hypothesis that IAA influences the regeneration of new thalli following decapitation in *C. braunii*. We found that IAA‐treated plants regenerated longer thalli, indicating the role of IAA in promoting growth. This is consistent with recent findings by Zeng *et al*. (2024), which showed that IAA enhanced thallus elongation within 6 h of treatment. However, while Zeng *et al*. (2024) reported that NPA inhibits elongation at the second and third internode, our study did not confirm this inhibitory effect. Moreover, we demonstrated that IAA promotes axillary branching at the basal node, in contrast to the typical auxin response in land plants, where auxin produced at the shoot apex suppresses lateral branching (Blake *et al*., 1983). However, shoot branching in land plants is regulated by a complex network of signals (Barbier *et al*., 2019), and any similarity between *Chara* branching and land plant shoot branching represents convergent evolution.

### *Chara* PINs are localized to the PM in vegetative and generative cells

The high rate at which exogenously applied IAA was depleted from the *Chara* biomass and medium suggested the presence of a highly efficient auxin homeostatic machinery, part of which was sensitive to auxin efflux inhibitor NPA. The genome of *C. braunii* contains no homologs of genes from canonical land plant auxin signaling and biosynthetic pathways (Nishiyama *et al*., 2018) but previously shown NPA‐sensitive auxin transport suggested the presence of carrier‐mediated auxin efflux (Boot *et al*., 2012). The PIN family of auxin efflux carriers is one of the most extensively studied membrane protein families in plants characterized by their ability to localize to distinct membrane compartments (Wiśniewska *et al*., 2006; Naramoto, 2017). While endomembrane‐localized noncanonical PINs are presumably involved in the regulation of intracellular auxin homeostasis (Mravec *et al*., 2009; Sauer & Kleine‐Vehn, 2019), the canonical, PM‐localized PINs play a crucial role in polar auxin transport, essential for plant growth and development (Luschnig & Friml, 2024). Moreover, the PIN homolog in the early‐diverging streptophyte *K. flaccidum* has been shown to be a functional auxin exporter (Skokan *et al*., 2019). The genome of *C. braunii* (Nishiyama *et al*., 2018) revealed six PIN homologs (*CbPINa* to *CbPINf*), which is remarkable as more than two paralogs have not been observed in any other streptophyte algae (Vosolsobě *et al*., 2020). This likely resulted from specific gene radiation in *Chara* (Nishiyama *et al*., 2018) possibly driven by transposable elements, as suggested by the presence of unusually long introns (> 20 kb) in its genome (Feng *et al*., 2024). We examined the structure and localization of two PIN homologs, CbPINa and CbPINc, which possess features similar to canonical PM‐localized PINs in land plants (Bennett *et al*., 2014). Their cell‐specific expression patterns suggest distinct roles, indicating potential involvement in *Chara* body plan development. Furthermore, some CbPINs might be needed in certain environmental conditions, as indicated by the upregulation of CbPINc under elevated salt concentrations (Heß *et al*., 2023a). Unfortunately, lack of a transformation system in *Chara* limited our ability to directly test these hypotheses *in vivo*.

The localization patterns of both CbPINs in the acid bands of internodal cells suggested an association with charasomes, the convoluted domains of *Chara* PM (Foissner *et al*., 2015), supported with a partial colocalization with PM H^+^‐ATPase. While the land plant PM H^+^‐ATPases are phosphorylated in response to auxin (Takahashi *et al*., 2012), *Chara* PM H^+^‐ATPases are insensitive to auxin (Zhang *et al*., 2016) and activated by light instead (Zeng *et al*., 2024). The acid bands, likely formed through the activity of PM H^+^‐ATPases, are regions of the cells involved in the uptake of dissolved inorganic carbon (Bulychev *et al*., 2001) and, in *Nitella*, are sites of cell elongation (Métraux *et al*., 1980). Therefore, the localization of CbPINs at the acid bands may reflect their role in auxin‐mediated cell elongation as shown in this study and in the study by Zeng *et al*. (2024). Although PIN auxin efflux carriers have been proposed to contribute to polar auxin transport in *Chara* (Boot *et al*., 2012), the thickness and impermeability of the *Chara* cell wall prevented us from successfully immunostaining intact thallus. We therefore cannot conclusively determine whether CbPINa and CbPINc exhibit polar localization in *C. braunii* during protonema germination or thallus development. Moreover, the presence of six PIN homologs suggests potential redundancy (Vieten *et al*., 2005; Janacek *et al*., 2024) or specialization that warrants further investigation. In antheridia, CbPINa was present in shield cells, while both CbPINs were localized more abundantly at the PM toward the ends of the filaments. This pattern contrasts with previous findings using heterologous antibodies against AtPIN2 in *C. vulgaris*, which showed more signal in capitular cells (Żabka *et al*., 2016). Furthermore, we found no evidence for a polar distribution of CbPINs in antheridial filament cells, further emphasizing the functional divergence between *Chara* PINs and their land plant counterparts.

### Unclear interference of CbPINs with auxin transport in heterologous systems

To assess their auxin transport capabilities, we inducibly expressed CbPINa and CbPINc in tobacco BY‐2 cells. In contrast to the single PIN from *Klebsormidium*, which mediates a clear auxin export, when expressed in BY‐2 cells (Skokan *et al*., 2019), we observed more ambiguous results with *Chara* PINs. Specifically, the induced PM‐localized CbPINa showed higher accumulation of [^3^H]‐NAA, whereas the CbPINc, which was mainly localized in the ER, did not significantly affect auxin accumulation. The lack of clear auxin transport may be due to several reasons: they may require specific interacting proteins or post‐translational modifications (e.g. phosphorylation) that are absent in BY‐2 cells; the extended hydrophilic loop of CbPINc may lead to improper folding when expressed heterologously, potentially resulting in an autoinhibitory effect; or the native lipid environment of *Chara* PM may be different from the environment in a heterologous system (Janacek *et al*., 2024). On the other hand, our auxin transport data supported by docking experiments indicated that CbPINa activity interferes with NPA‐sensitive [^3^H]‐NAA auxin efflux, and that it interacts extensively with IAA, NAA, and NPA. CbPINa may theoretically bind auxin, but not to transport it. In this case, overexpression of CbPINa would result in the higher accumulations of [^3^H]‐NAA, the opposite to its decreased accumulation, when transporting PINs from *Arabidopsis* or tobacco are overexpressed (Petrášek *et al*., 2006; Müller *et al*., 2019). The binding but not transporting of auxin has been recently shown for LYCHOS (GPR155), a human homolog of PIN auxin efflux carriers (Bayly‐Jones *et al*., 2024).

We further extended our investigation to land plant models *A. thaliana* and *M. polymorpha*. In *Arabidopsis*, both CbPINs showed signal in the ER and PM, but unlike the AtPIN2, they did not localize polarly and failed to rescue the *pin2* mutant, indicating functional divergence from land plant PINs. In *M. polymorpha* gemmalings, CbPINa predominantly localized to the PM, as confirmed by a PM marker, but failed to rescue the described Mp*pin1* mutant phenotype (Fisher *et al*., 2023). These findings further support that CbPINa localizes predominantly to the PM in both heterologous and homologous systems, while CbPINc is primarily ER‐localized in heterologous systems but PM‐localized in *Chara*.

### Rapid responses to IAA and its perception in *Chara*

While the canonical, nuclear auxin pathway is widely considered to be a unique feature of land plants that emerged only during terrestrialization (Hernández‐García *et al*., 2024), recent discoveries of a rapid auxin response dependent on cell surface auxin perception (Friml *et al*., 2022) conserved in both land plants and streptophyte algae suggest that this auxin signaling pathway is more ancient (Kuhn *et al*., 2024). Our analysis of phosphoproteome, coupled with the analysis of cytoplasmic streaming in *Chara* branchlet internodal cells, revealed distinct auxin‐triggered phosphorylation patterns. These include candidates such as the *Arabidopsis* mitogen‐activated protein kinase (MAP4K class III) homolog (Pan *et al*., 2021), a sodium/hydrogen exchanger homologous to NHX5 and NHX6, which are essential for PIN6‐mediated auxin homeostasis in *Arabidopsis* (Lv *et al*., 2020), and a RAF‐like kinase homolog (Kuhn *et al*., 2024) all of which support the deeply conserved role of auxin in rapid cellular responses. However, the number of significantly hyper‐ and hypophosphorylated phosphosites was smaller than in previously reported species (Kuhn *et al*., 2024), likely due to the limited coverage of the *Chara* genome or due to the heterogeneity of samples.

### Conclusion

Taken together, our findings have revealed both growth promoting and fast auxin effects in *Chara*. While immunostaining of CbPINa and CbPINc indicated plasma membrane association, their role in auxin‐mediated transport in *Chara* has yet to be determined. Future research should investigate the remaining four PIN homologs and molecular components of the rapid auxin signaling in *Chara* to gain additional insights into the evolution of plant morphological complexity.

## Competing interests

None declared.

## Author contributions

KK, SV and JP conceptualized the study. KK performed *C. braunii* experiments that included cultivation, harvesting, immunolocalization, and growth responses, cloned *Chara* PINs, transformed BY2 and performed western blot. PP performed immunolocalization of tubulin in *Chara*. SV computed RNA‐seq results, performed statistical analysis of growth experiments, cytoplasmic streaming and STRING analysis. KM isolated RNA from *Chara* and performed RT‐qPCR. DN performed auxin accumulation assays and *in silico* PIN docking. PID measured IAA metabolism. VS analyzed metabolism data. AK performed isolation of proteins for phosphoproteomics, phosphoenrichment and phosphoproteomic analysis. AS performed *A. thaliana* experiments. TJF performed *M. polymorpha* experiments. KK, SV and JP wrote the manuscript. DW, JF and JLB revised the manuscript.

## Disclaimer

The New Phytologist Foundation remains neutral with regard to jurisdictional claims in maps and in any institutional affiliations.

## Supporting information


**Fig. S1** Design of cultivation box for *Chara braunii*.
**Fig. S2** Auxin treatment experiment and plasma membrane staining in axillary branches of *Chara braunii*, strain NIES 1604.
**Fig. S3** Concentration of indole‐3‐acetic acid (IAA) and IAA metabolites in *Chara braunii* biomass and its medium determined by LC‐MS.
**Fig. S4** Melting curve analysis from RT‐qPCR and expression profiles of *Chara braunii* PINs during life cycle.
**Fig. S5** Negative controls for CbPINa and CbPINc immunostainings.
**Fig. S6** Positive controls for CbPINa and CbPINc immunostainings.
**Fig. S7** Auxin transport assays in tobacco BY‐2 cells.
**Fig. S8** Multiple sequence alignment showing conserved sites between *Arabidopsis thaliana*, *Marchantia polymorpha*, and *Chara braunii* PINa and PINc.
**Fig. S9** STRING analysis of significantly phosphorylated candidates upon indole‐3‐acetic acid treatment.
**Methods S1** Construction of cultivation box for *Chara braunii*.
**Methods S2** Immunolocalization of internodal cells with CbPINs and H^+^ATPase.
**Methods S3** Immunolocalization of antheridia CbPINs and H^+^ATPase.
**Methods S4** Immunolocalization of tubulin in internodal cells.
**Methods S5** Immunolocalization of tubulin in antheridial cells.
**Methods S6** Protein extraction and western blot.
**Methods S7** Sample preparation for phosphoproteomic analysis.
**Methods S8** Protein extraction for phosphoproteomic analysis.
**Methods S9** Phosphopeptide enrichment.
**Methods S10** Statistical analysis.
**Table S1** List of chemicals and components.
**Table S2** List of primers.
**Table S3** PIN‐FORMED auxin efflux carriers in *Chara braunii*.
**Table S4** Ligand biding affinities of CbPINs with indole‐3‐acetic acid, 1‐NAA, and N‐1‐naphthylphthalamic acid calculated in Autodock.
**Table S5** Significantly phosphorylated proteins under indole‐3‐acetic acid treatment compared with dimethyl sulfoxide.
**Table S6** Significantly dephosphorylated proteins under indole‐3‐acetic acid treatment compared with dimethyl sulfoxide.

**Video S1** Cytoplasmic streaming of branchlet internodal cells after dimethyl sulfoxide treatment.

**Video S2** Cytoplasmic streaming of branchlet internodal cells after 0.1 μM indole‐3‐acetic acid.

**Video S3** Cytoplasmic streaming of branchlet internodal cells after 1 μM indole‐3‐acetic acid.

**Video S4** Cytoplasmic streaming of branchlet internodal cells after 0.1 μM benzoic acid.

**Video S5** Cytoplasmic streaming of branchlet internodal cells after 1 μM benzoic acid.
Please note: Wiley is not responsible for the content or functionality of any Supporting Information supplied by the authors. Any queries (other than missing material) should be directed to the *New Phytologist* Central Office.

## Data Availability

The data that supports the findings of this study are openly available on Zenodo repository at doi: 10.5281/zenodo.14040634. Phosphoproteome raw data are available via ProteomeXchange with identifier PXD059774. Complete source codes are deposited at https://github.com/vosolsob/chara. The updated sequence of CbPINa is available on GenBank with accession no. PQ421084.
