# Supplementary material for: The role of indole‐3‐acetic acid and characterization of PIN transporters in complex streptophyte alga Chara braunii
Source: New Phytol. 2025 Mar 6;246(3):1066–83. doi: 10.1111/nph.70019 (PMC11982790; doi:10.1111/nph.70019)
Supplement: Supplementary file 1 — Fig. S1 Design of cultivation box for Chara braunii. Fig. S2 Auxin treatment experiment and plasma membrane staining in axillary branches of Chara braunii, strain NIES 1604. Fig. S3 Concentration of indole‐3‐acetic acid (IAA) and IAA metabolites in Chara braunii biomass and its medium determined by LC‐MS. Fig. S4 Melting curve analysis from RT‐qPCR and expression profiles of Chara braunii PINs during life cycle. Fig. S5 Negative controls for CbPINa and CbPINc immunostainings. Fig. S6 Positive controls for CbPINa and CbPINc immunostainings. Fig. S7 Auxin transport assays in tobacco BY‐2 cells. Fig. S8 Multiple sequence alignment showing conserved sites between Arabidopsis thaliana, Marchantia polymorpha, and Chara braunii PINa and PINc. Fig. S9 STRING analysis of significantly phosphorylated candidates upon indole‐3‐acetic acid treatment. Methods S1 Construction of cultivation box for Chara braunii. Methods S2 Immunolocalization of internodal cells with CbPINs and H+ATPase. Methods S3 Immunolocalization of antheridia CbPINs and H+ATPase. Methods S4 Immunolocalization of tubulin in internodal cells. Methods S5 Immunolocalization of tubulin in antheridial cells. Methods S6 Protein extraction and western blot. Methods S7 Sample preparation for phosphoproteomic analysis. Methods S8 Protein extraction for phosphoproteomic analysis. Methods S9 Phosphopeptide enrichment. Methods S10 Statistical analysis. Table S1 List of chemicals and components. Table S2 List of primers. Table S3 PIN‐FORMED auxin efflux carriers in Chara braunii. Table S4 Ligand biding affinities of CbPINs with indole‐3‐acetic acid, 1‐NAA, and N‐1‐naphthylphthalamic acid calculated in Autodock. Table S5 Significantly phosphorylated proteins under indole‐3‐acetic acid treatment compared with dimethyl sulfoxide. Table S6 Significantly dephosphorylated proteins under indole‐3‐acetic acid treatment compared with dimethyl sulfoxide. [file NPH-246-1066-s005.pdf]

New Phytologist **Supporting Information**

Article title: The role of IAA and characterization of PIN transporters in complex streptophyte alga *Chara braunii*

Authors: Katarina Kurtović, Stanislav Vosolsobě, Daniel Nedvěd, Karel Müller, Petre Ivanov Dobrev, Vojtěch Schmidt, Piotr Piszczek, Andre Kuhn, Adrijana Smoljan, Tom J. Fisher, Dolf Weijers, Jiří Friml, John L. Bowman, Jan Petrášek

Article acceptance date: 23 January 2025

The following Supporting Information is available for this article:

Fig. S1 Design of cultivation box for *Chara braunii*.

Fig. S2 Auxin treatment experiment and plasma membrane staining in axillary branches of *Chara braunii*, strain NIES 1604.

Fig. S3 Concentration of IAA and IAA metabolites in *Chara braunii* biomass and its medium determined by LC/MS.

Fig. S4 Melting curve analysis from RT-qPCR of and expression profiles of *Chara braunii* PINs during life cycle.

Fig. S5 Negative controls for CbPINa and CbPINc immunostainings.

Fig. S6 Positive controls for CbPINa and CbPINc immunostainings.

Fig. S7 Auxin transport assays in tobacco BY-2 cells.

Fig. S8 Multiple sequence alignment showing conserved sites between *Arabidopsis thaliana*, *Marchantia polymorpha*, and *Chara braunii* PINa and PINc.

Fig. S9 STRING analysis of significantly phosphorylated candidates upon IAA treatment.

Table S1 List of chemicals and components.

Table S2 List of primers.

Table S3 PIN-FORMED auxin efflux carriers in *Chara braunii*.

Table S4 Ligand biding affinities of CbPINs with IAA, 1-NAA, and NPA calculated in Autodock.

Table S5 Significantly phosphorylated proteins under IAA treatment compared to DMSO.

Table S6 Significantly dephosphorylated proteins under IAA treatment compared to DMSO.

Methods S1 Construction of cultivation box for *Chara braunii.*

Methods S2 Immunolocalization of internodal cells with CbPINs and H^+ATPase.

Methods S3 Immunolocalization of antheridia CbPINs and H^+ATPase.

Methods S4 Immunolocalization of tubulin in internodal cells.

Methods S5 Immunolocalization of tubulin in antheridial cells.

Methods S6 Protein extraction and Western blot.

Methods S7 Sample preparation for phosphoproteomic analysis.

Methods S8 Protein extraction for phosphoproteomic analysis.

Methods S9 Phosphopeptide enrichment.

Methods S10 Statistical analysis.

Video S1 Cytoplasmic streaming of branchlet internodal cells after DMSO treatment.

Video S2 Cytoplasmic streaming of branchlet internodal cells after 0.1 µM IAA.

Video S3 Cytoplasmic streaming of branchlet internodal cells after 1 µM IAA.

Video S4 Cytoplasmic streaming of branchlet internodal cells after 0.1 µM BA.

Video S5 Cytoplasmic streaming of branchlet internodal cells after 1 µM BA.

(videos attached as separate files)

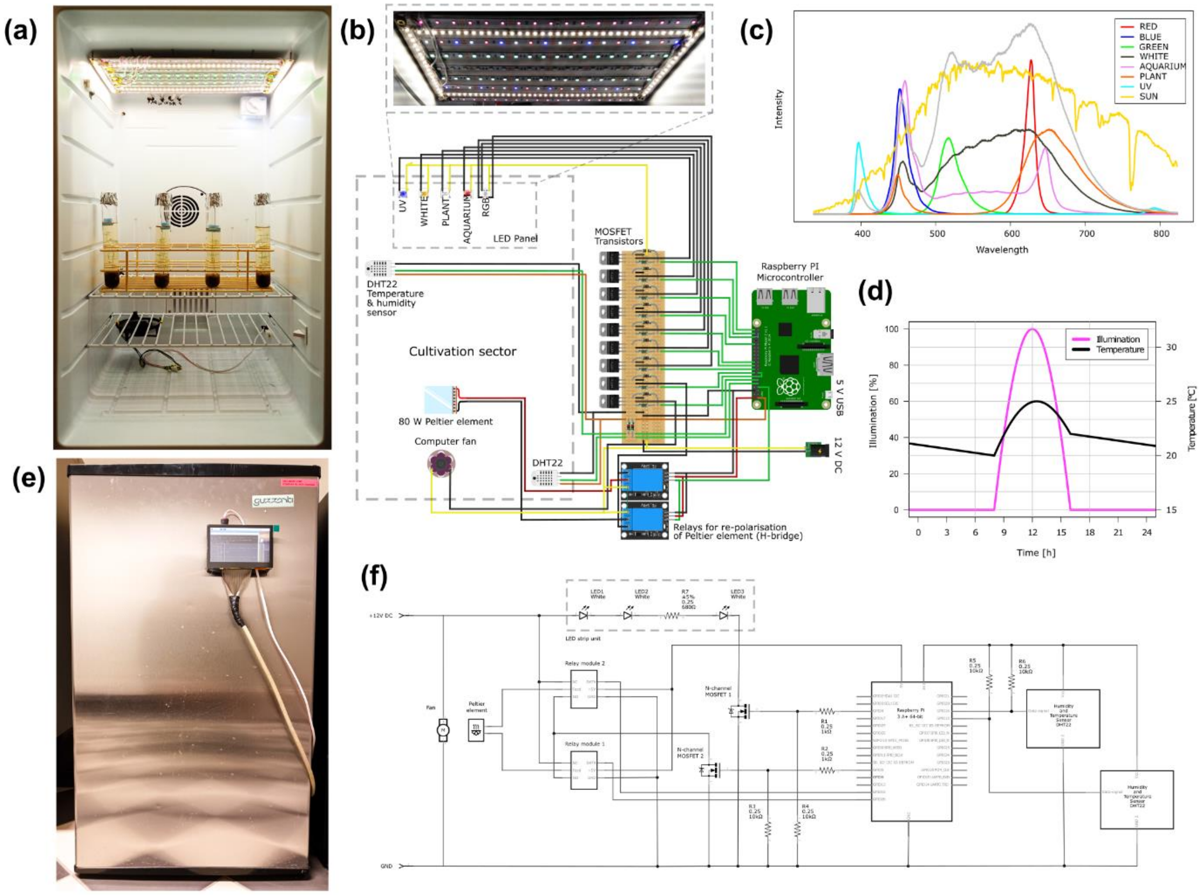

Fig. S1 Design of cultivation box for *Chara braunii*. (a,e) Commercial thermoelectric refrigerator is equipped with a custom LED panel **(b)** consisting of RGB, day white, and UV LED strips, as well as special LED strips designed for aquariums and plant growth with extended far-red emission. Below LED strips is shown regulation of one type of LED stripe and regulation of the Peltier element via H-bridge and MOSFET, which is constructed done with respect to the actual temperature measured by a pair of DHT22 sensors. Built Built-in PC fan ensures homogenization of the temperature inside the box. **(c)** The sun spectrum is shown for comparison. The gray line shows an example of the LEDs' intensity setup with maximal similarity to natural sunlight. **(d)** Exemplary day-courses of illumination and temperature showing non-linear simulation of those environmental factors, close to natural situation **(e)** Both illumination and temperature in the box is regulated by MOSFET transistors and the Raspberry PI microcomputer placed on the fridge's door together with touch display enabling the checking of internal parameters, which are controlled by a python script. **(f)**. Simplified wiring diagram.

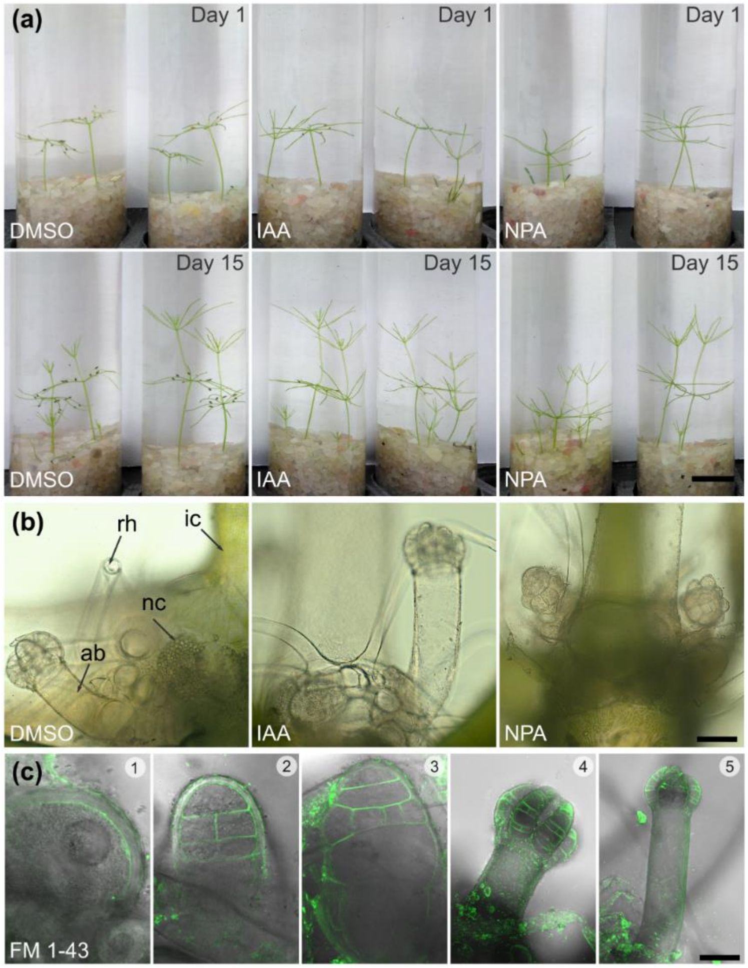

Fig. S2 Auxin treatment experiment and plasma membrane staining in axillary branches of *Chara braunii*, strain NIES 1604. (a) A representative thallus regeneration experiment with DMSO (control), 1 µM IAA, and 10 µM NPA treatments. The decapitated explants used for regeneration consisted of two nodal complex cells and one internodal cell between them. The bottom nodal complex is sown underneath the sand surface to initiate the rhizoid formation, while the apical nodal regenerates a new thallus **(b)** Brightfield images of axillary branches at the basal node. The node is underneath the surface of the medium, therefore branches that develop underground are transparent. When they are exposed to light, they develop chloroplasts and become photosynthetic. Rhizoids (rh), internodal cell (ic), axillary branch (ab), nodal cell (nc). **(c)** FM 1-43 staining of PM in various stages (1-5) of axillary branches that developed underground. Since these organs develop without light, the plasma membrane is smooth compared to the invaginated plasma membrane of internodal cells that form charasomes in response to light.

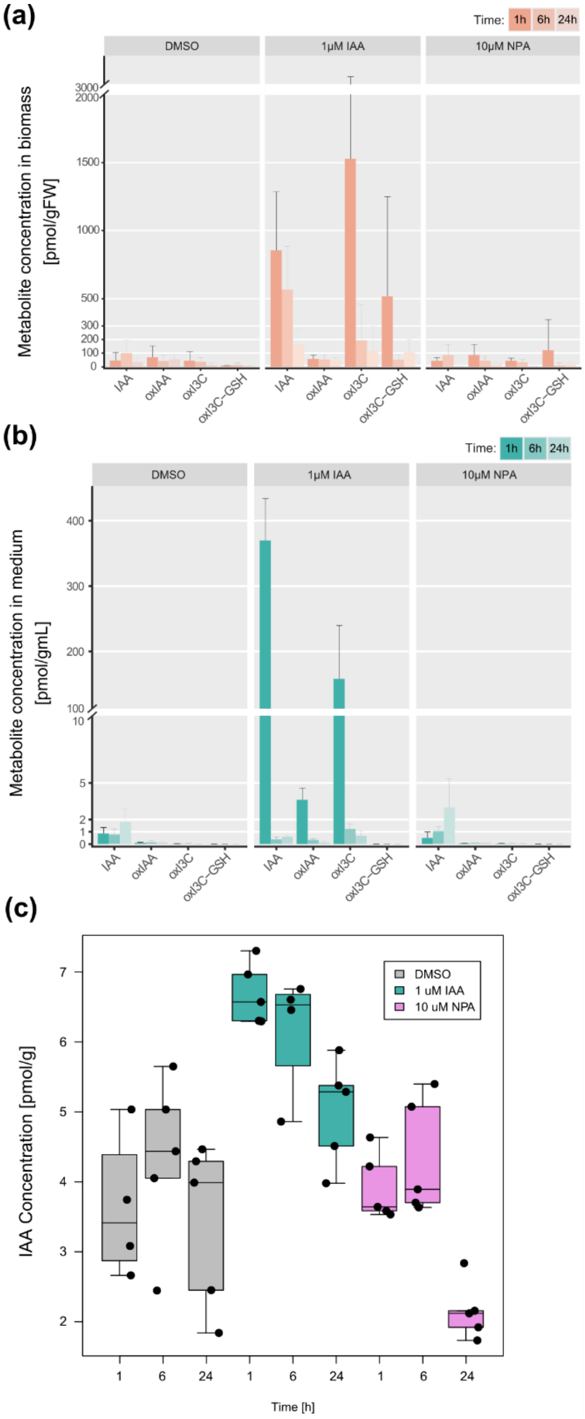

Fig. S3 Concentration of IAA and IAA metabolites in *Chara braunii* determined by LC/MS. (a) Metabolite concentration in 3-week-old *Chara braunii* biomass The treatments included mock (DMSO), 1 µM IAA and 10 µM NPA. Samples were collected after 1h, 6h, and 24h. 2-oxindole-3-acetic acid (oxIAA), oxindole-3-carbinol (oxI3C), oxindole-3-carbinol-gluthathione (oxI3C-GSH). Error bars show ±SD. **(b)** Metabolite concentration in corresponding medium in which *Chara*

braunii thalli were grown and treated with mock (DMSO), 1 µM IAA, and 10 µM NPA. Biomass collection was followed immediately followed by sampling of the liquid medium. Error bars show ±SD. **(c)** Boxplot showing concentration of IAA in *Chara braunii* biomass expressed as logarithmic scale. Note the very low concentration of IAA after 24 h treatment compared to 24h of DMSO treatment. Medians, first and third quartiles, and 95% confidence intervals of medians are shown.

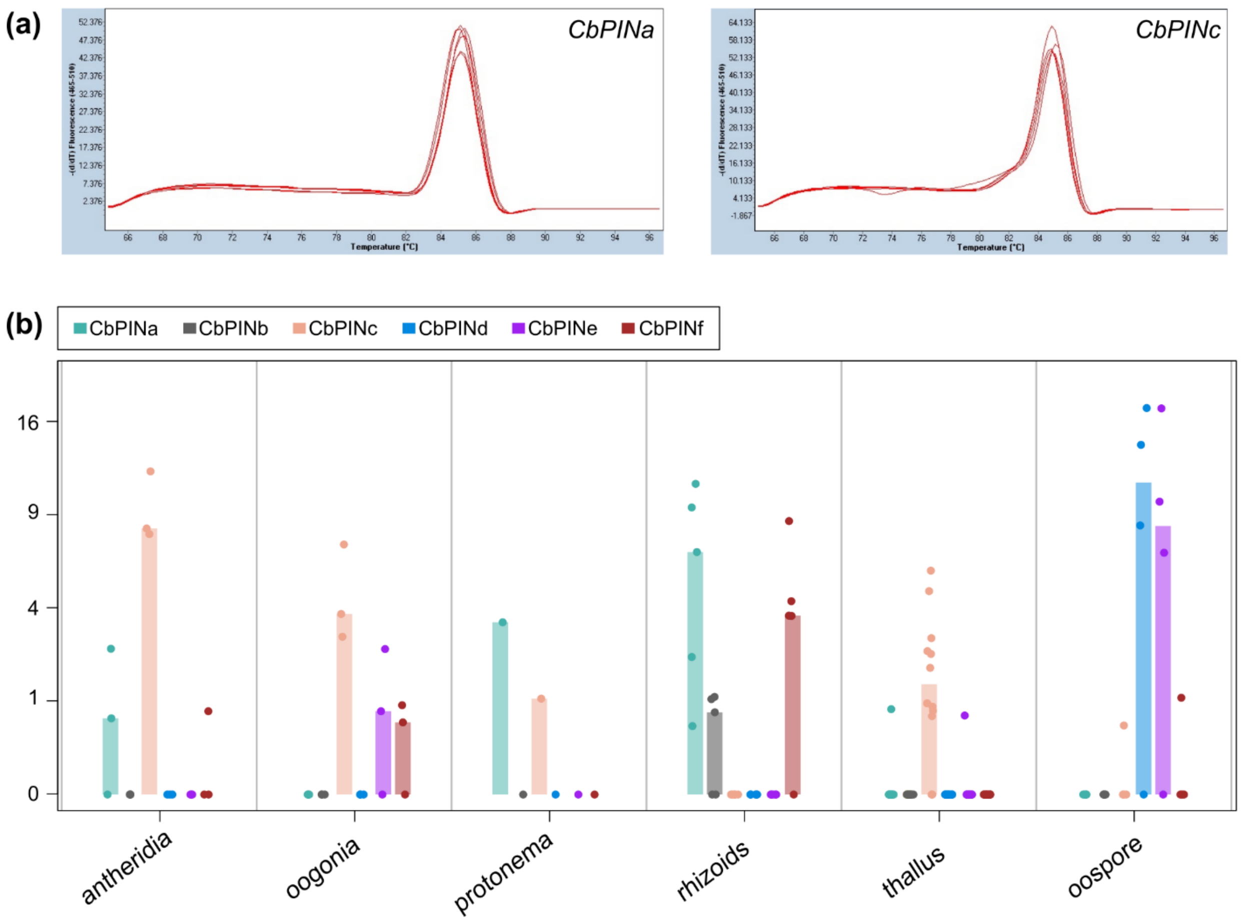

Fig. S4 Melting curve analysis from RT-qPCR of and expression profiles of *Chara braunii* PINs during the life cycle. (a) Melt curves from RT-qPCR of *CbPINa* and *CbPINc*. The dissociation temperatures range from 65°C to 96°C. Amplicons from both *CbPINa* and CbPINc reveal a single peak. **(b)** Data from publicly available RNA-seq libraries (Datasets) were quantified by Kallisto and STAR. Not**e** higher expressions of *CbPINa* in protonema and rhizoids, while *CbPINc* is expressed in all stages except rhizoids and oospore. The y-axis show transcripts per million. (*TPM*). The threshold of *TPM* > 0.5 was applied when the results were visualized in R.

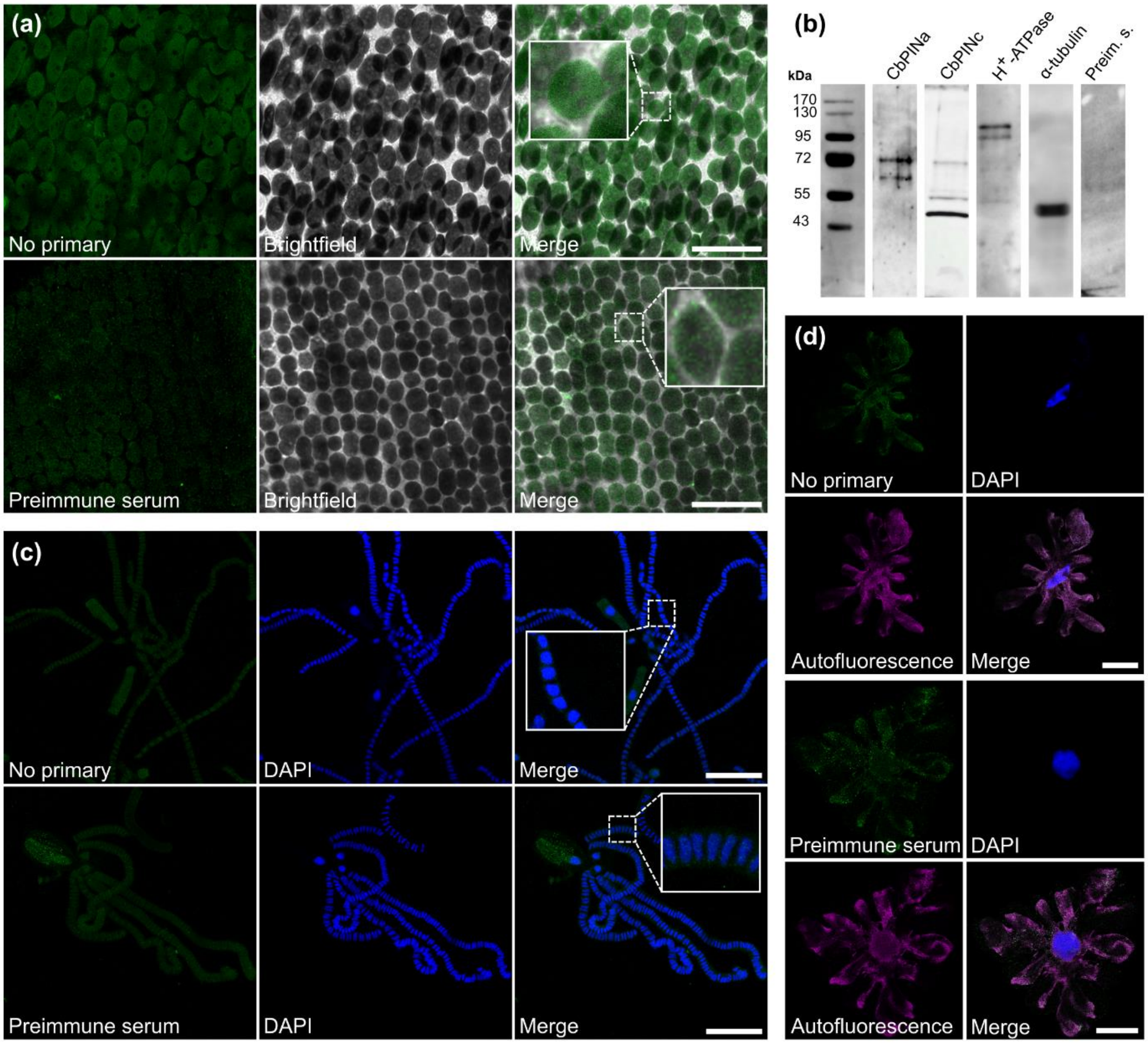

Fig. S5 Negative controls for CbPINa and CbPINc immunostainings. (a) Omission of primary antibody and preimmune serum staining in internodal cells. **(b)** Western blots of CbPINa, CbPINc, H^+-ATPase, α-tubulin, and preimmune serum. The preimmune serum that served as a negative control shows no visible band on the gel. CbPINa is recognized by a band around 70 kDa which corresponds to the approximate size of the protein, while CbPINc shows multiple bands, likely resulting from partial cleavage of the 110 kDa protein. The cleavage sites could occur between the epitopes, leading to multiple fragments that include the transmembrane domain, transmembrane domain with portion of the loop, and the loop region of the protein. **(c)** Omission of primary antibody and preimmune serum staining in antheridial filaments. **(d)**

Omission of primary antibody, preimmune serum staining, and autofluorescence in antheridia shield cells. Scale bars, 20 µm (a) 50 µm **(b, c, d)**.

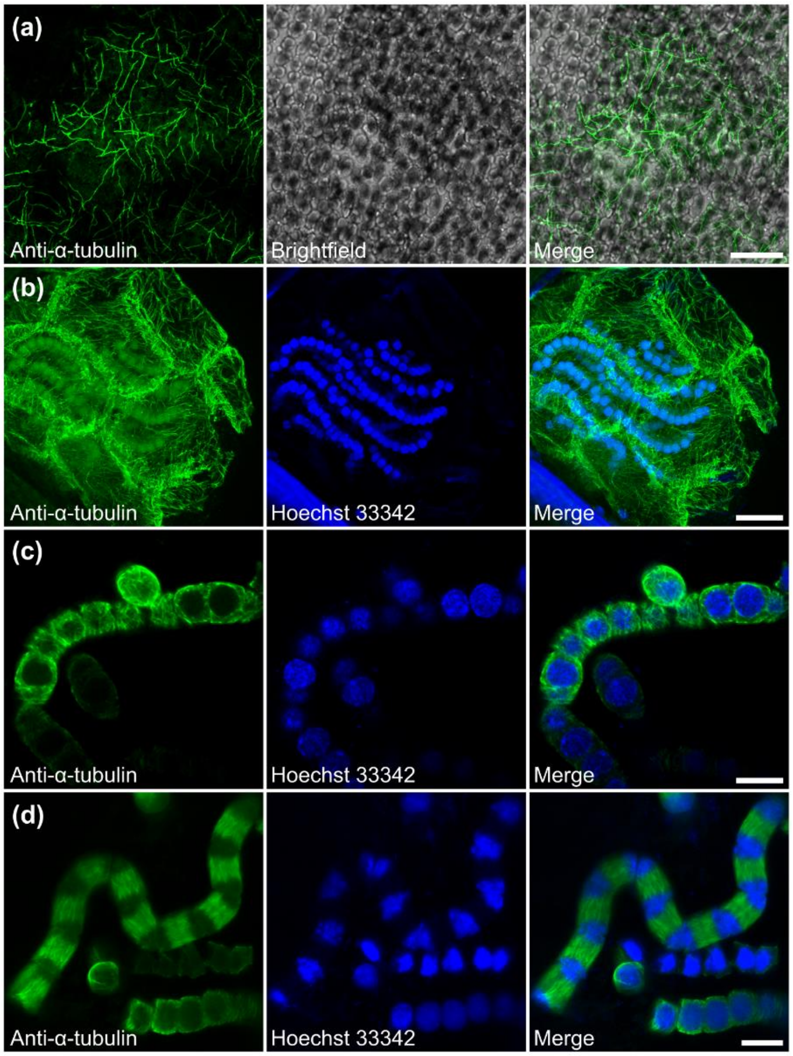

Fig. S6 Positive controls for CbPINa and CbPINc immunostainings performed by anti-α-tubulin staining. (a) The internodal cell treated with taxol, confocal, brightfield, and merged image. **(b)** Maximal projection of the whole antheridium. Shield cells are stained with anti-α-tubulin in green, blue Hoechst-stained nuclei of antheridial filaments, and a merged image. **(c)** Free antheridial filaments showing α-tubulin, Hoechst staining, and a merged image. **(d)** Antheridial filament during the cell division. Scale bars, 20 µm **(a, b),** 10 µm **(c, d).**

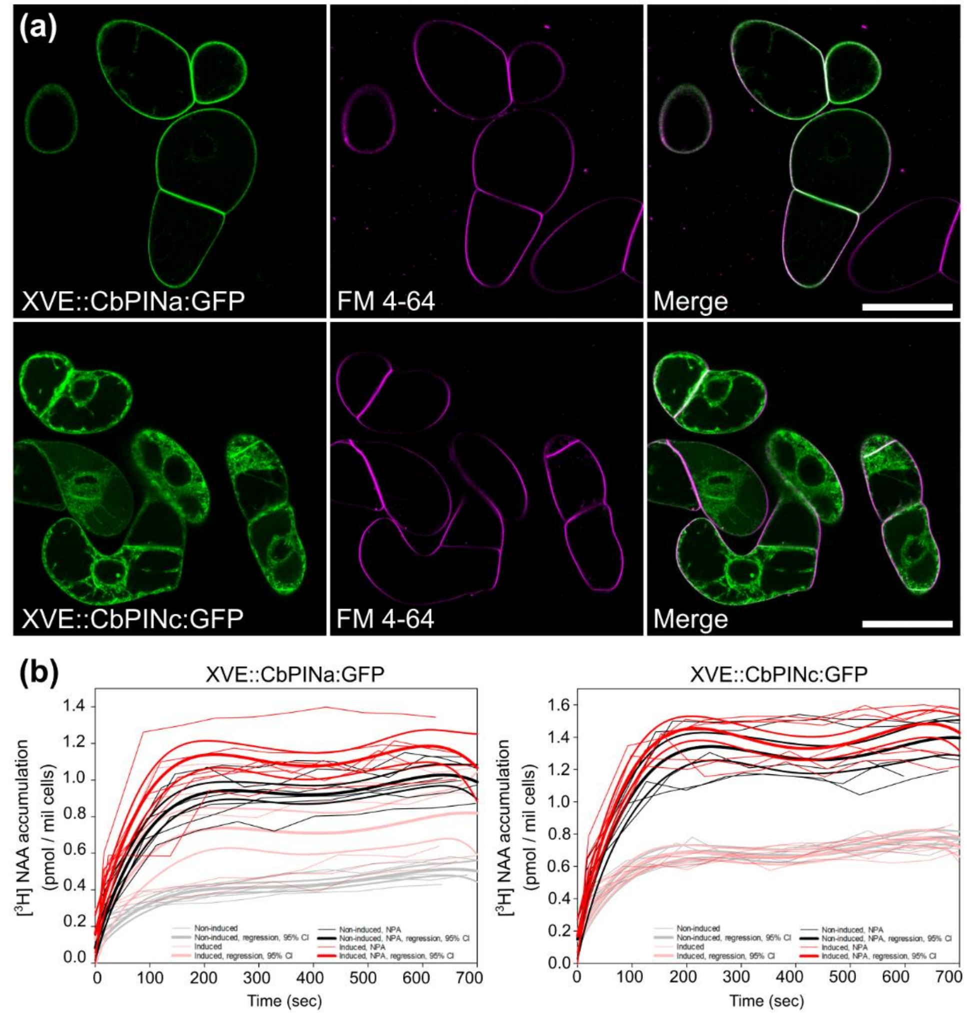

Fig. S7 Auxin transport assays in tobacco BY-2 cells. **(a)** Induced tobacco BY-2 cells expressing XVE:CbPINa:GFP and XVE:CbPINc:GFP. The GFP signal at the PM and endoplasmic reticulum, FM 4-64 PM staining and merged images are shown. **(b)** Kinetics of [^{3}H]-NAA accumulation in 5-day-old induced and non-induced XVE:CbPINa:GFP and XVE:CbPINc:GFP BY-2 cells, NPA (10 μM). The x-axis contains the length of the accumulation run in seconds, while y-axis shows the amount of radioactively labeled NAA expressed in pmol/mil cells. The thin lines represent independent accumulation runs (*n* = 6, 3 biological repeats, each with two technical repeats), and thick lines represent the smoothed average.

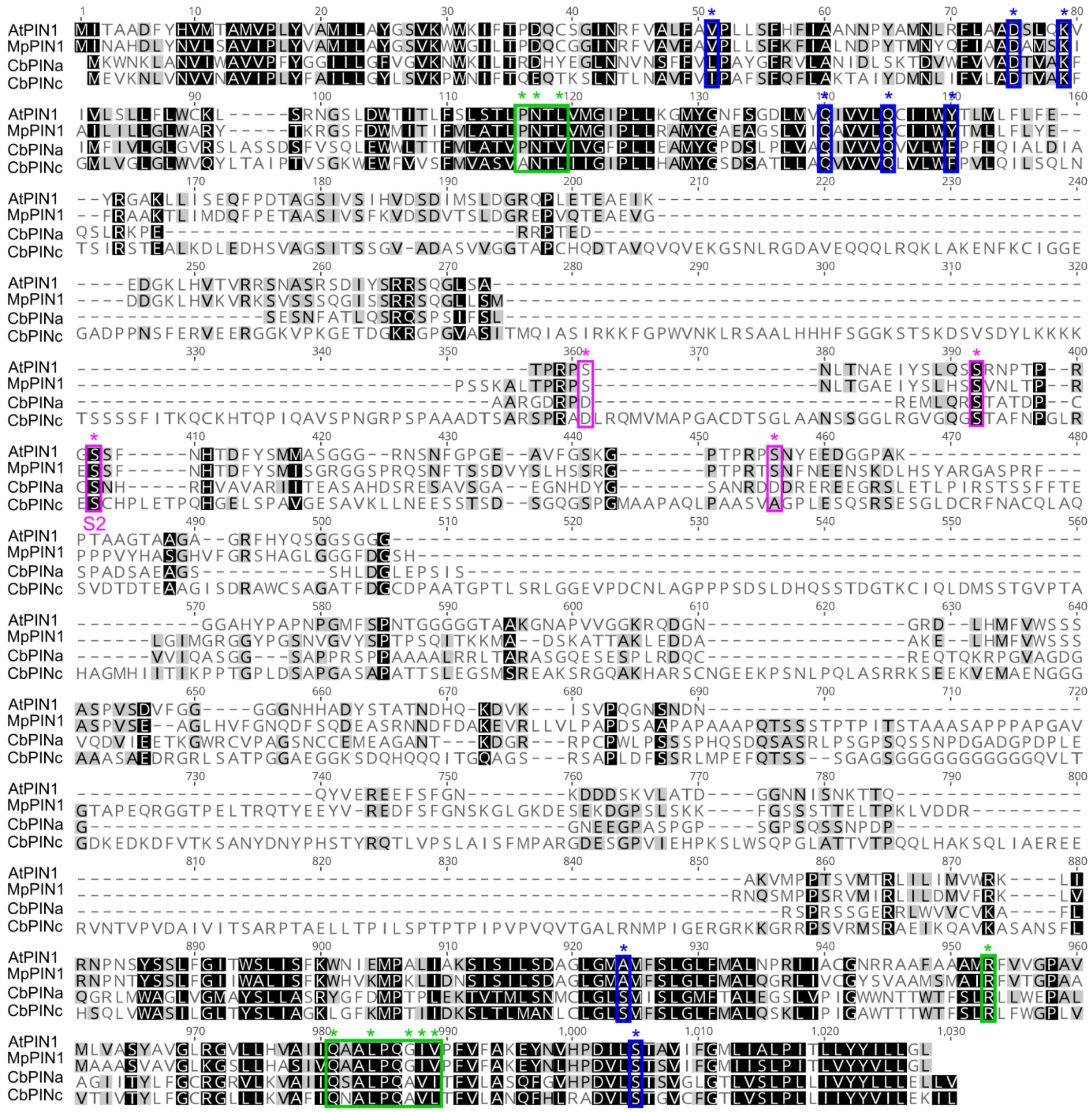

Fig. S8 Multiple sequence alignment showing conserved sites between *Arabidopsis thaliana*, *Marchantia polymorpha,* and *Chara braunii* PINa and PINc. The potential phosphorylation sites are framed in magenta. The serine residues that could be potentially phosphorylated are marked with an asterisk, note the S2 phosphorylation site. The green frames represent the sites in TM4 and TM9 that are responsible for the crossover mechanism, and the conserved arginine in TM8 that stabilizes dimerization. Blue frames represent conserved sites that were described to additionally facilitate the auxin transport.

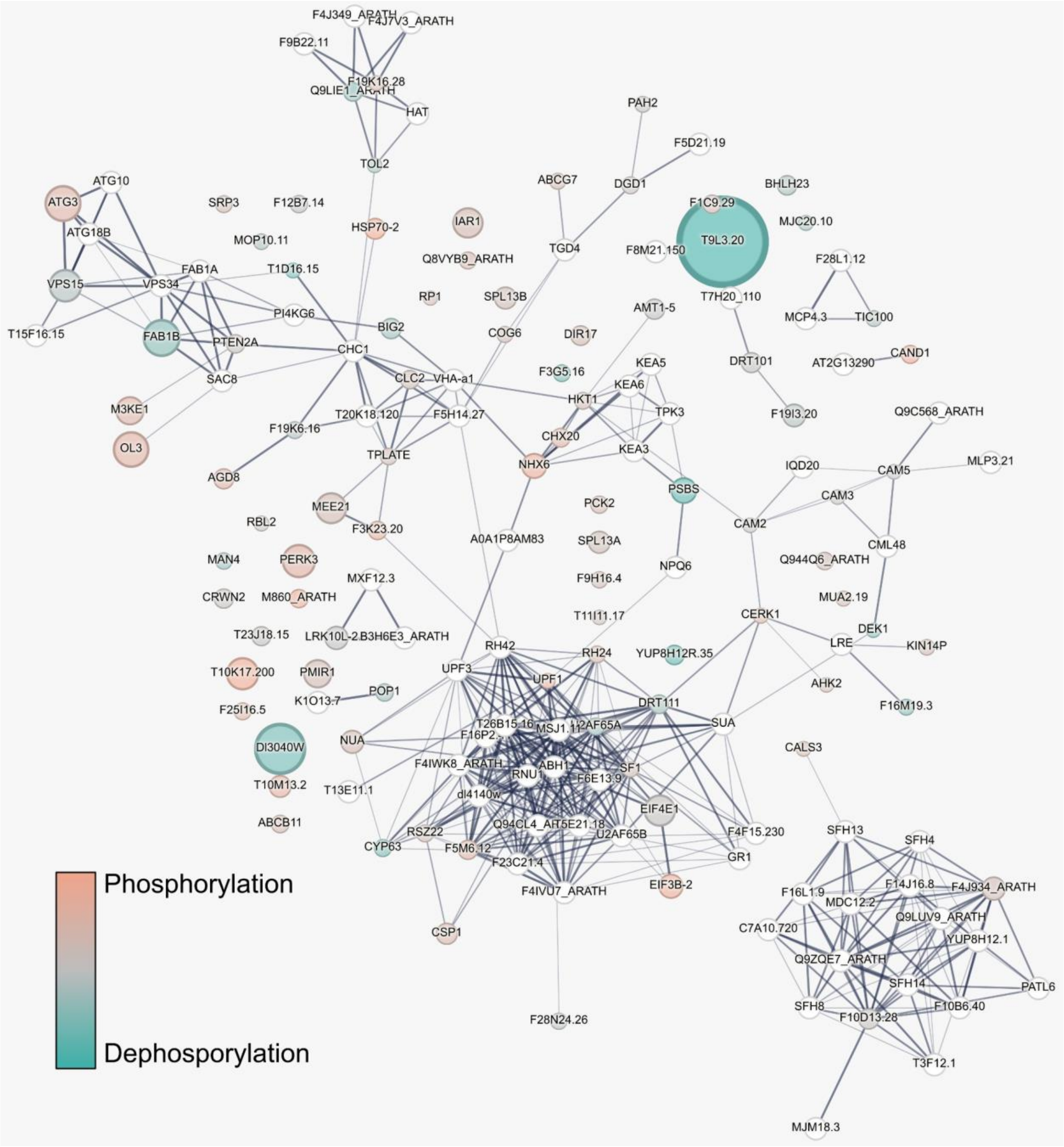

Fig. S9 STRING analysis of significantly hyper-/dephosphorylated candidates upon 2-min IAA treatment. Network nodes represent the *A. thaliana* closest homologs to the identified candidates in *Chara braunii*. The color scale is "blue-gray-orange" shows the rate of change (phosphorylation/dephosphorylation), the diameter of circles is proportional to significance log (*p-value*) of *t*-test. Second-shell interactors added to the STRING network were shown by empty circles.

Table S1 List of chemicals and components.

REAGENT or RESOURCE	SOURCE	IDENTIFIER
DNeasy Plant Mini Kit	QIAGEN	Cat# 69104
Q5® High-Fidelity DNA Polymerase	New England Biolabs	Cat# M0491S
Q5® Reaction Buffer	New England Biolabs	Cat# B9027S
dNTP Set	Thermo Scientific™	Cat# R0181
XhoI	New England Biolabs	Cat# R0146S
DreamTaq DNA Polymerase	Thermo Scientific™	Cat# EP0701
DreamTaq™ Buffer (10X)	Thermo Scientific™	Cat# B65
SpeI-HF®	New England Biolabs	Cat# R3133S
AscI	New England Biolabs	Cat# R0558S
rCutSmart™ Buffer	New England Biolabs	Cat# B6004S
T4 ligase	New England Biolabs	Cat# M0202S
T4 buffer	New England Biolabs	Cat# B0202S
pJET1.2/blunt Cloning Vector	Thermo Scientific™	Cat# K1231
GelRed® Nucleic Acid Gel Stain	Biotium	Cat# 41003
Agarose	GIBCO BRL	Cat# 15510-027
1kb Ladder	Thermo Scientific™	Cat# SM0311
Gel Loading Dye, Purple (6X)	New England Biolabs	Cat# B7024S
DMSO	MP Biomedicals	Cat# 196055
Indole-3-acetic acid IAA	Merck	Cat# 45533
N-1-naphthylphthalamic acid (NPA)	OlChemIm s.r.o	Cat# 018 3043
Benzoic acid	Fluka	Cat# 12349
Glutaraldehyde	Merck	Cat# 111-30-8
Plant agar	Duchefa Biochemie	Cat# 9002-18-0
Slilica/glass sand (SiO2 content approx. 99%)	Provodínské písky a.s.	by order
Compost	Charles University, Faculty of Science, Botanical garden	

Perlit Agro	Hornbach	Cat# 4286915
Garden substrate	Hawita	Cat#
Glass tubes	Technosklo	Cat# 2800
**COMPONENT**	**SUPPLIER**	**IDENTIFIER**
Thermocooler Guzzanti GZ 70S	MALL.CZ	Cat# 1717017
Raspberry PI 3 A+ 64-bit 512MB RAM	RPishop.cz	Cat# RPI304
Raspberry Pi 32GB microSDHC Class 10 UHS-I U1 A1 with NOOBS and Raspbian	RPishop.cz	Cat# OFI065
Waveshare 4,3" DSI LCD display 800×480	RPishop.cz	Cat# WAV135
Aluminum heatsink 27.8x27.8x 11.2 mm	RPishop.cz	Cat# HTS011
PC Power supply ATX-350W	Upcycling from PC	
PC Fan 12V 120 mm	Upcycling from PC	
DHT22 temperature and humidity sensor	Drátek.cz	Cat# 1455993178
2-channel relay module - 5V input	Drátek.cz	Cat# 1500636006
LED strip TLP-300SMD-RGB 14.4 W.m-1	T-LED s.r.o.	Cat# 08210
LED strip CRI-600-DW 20 W.m-1	T-LED s.r.o.	Cat# 07504
LED strip UV5-300 14.4 W.m-1	T-LED s.r.o.	Cat# 07803
LED strip 12GROW 6012-FULL 12 W.m-1	T-LED s.r.o.	Cat# 078081
LED strip 12AKVA 6012-WBR 12 W.m-1	T-LED s.r.o.	Cat# 078080
MOSFET INFINEON IRLB3034PBF	GM-electronics	Cat# 213-362
Resistor 10K - 0,25W 1%	Drátek.cz	Cat# 1557994916
Resistor 1K - 0,25W 1%	Drátek.cz	Cat# 1557994890

Table S2 List of primers.

Primer name	Sequence
Chara braunii PIN cloning	
pJET1.2 Forward Sequencing Primer	CGACTCACTATAGGGAGAGCGGC
pJET1.2 Reverse Sequencing Primer	AAGAACATCGATTTTCCATGGCAG
pER8_P50 Forward Sequencing Primer	ATGCTCGACTCTAGGATCTTC
pER8_T50 Reverse Sequencing Primer	TCACACTGTTAGTTCCTTTCTTAC
PINa forward	ATGAAGTGGAACAAGCTTGCGAAC
PINa reverse	TCACACCAGGAGCTCTAATAATAGG
PINc 1st exon forward	ATGGAGGTCAAGAACCTGGTC
PINc 1st exon reverse	CAGAATCGAGAGGACCTGTTGG
CbPINc_1/2_F forward	CCAACAGCACATGCAGGAATGCACATCATC
CbPINc_1/2_R reverse	GATGATGTGCATTCCTGCATGTGCTGTTGG
1_AscICbPINa forward	CAGGCGCGCCATGAAGTGGAACAAGCTTGCGAAC
2_GFP_CbPINa reverse	GTTCTTCTCCTTTACCCATCCCTTCTAGCGGATCAG
3_CbPINa_GFP forward	ATCCGCTAGAAGGGATGGGTAAAGGAGAAG
4_CbPINa_GFP reverse	GCTGGTCCTTCTTCATTTCCGTATAGTTCATCCATGCC
5_GFP_CbPINa forward	GGCATGGATGAACTATACGGAAATGAAGAAGGACCAG
6_SpeI_CbPINa reverse	CTACTAGTTCACACCAGGAGCTCTAATAATAGG
CbPINc_AscI_F1 forward	GGCGCGCCATGGAGGTCAAGAAC
CbPINc_GFP_F2_long forward	CCAACAGCACATGCAATGGGTAAAGGAGAAG
CbPINc_GFP_F3 forward	GGATGAACTATACGGAATGCACATC
CbPINc_GFP_R1 reverse	TTACCCATTGCATGTGCTGTTGG
CbPINc_GFP_R2_long reverse	GATGTGCATTCCGTATAGTTCATCCATGCC
CbPINc_SpeI_R3 reverse	GTGCTGCATCTTGAGTGACGACTAGT
RT-qPCR	
ChEF1a_qFor1	GGCGAACGGGTAAGGAAATC
ChEF1a_qRev1	GATACTCGGCGAAGGTCTCA
ChPINa_qFor1	TTGATATGCCGACGCCTTTG
ChPINa_qRev1	GGGACCAAACTGCCTTCAAG
ChPINc_qFor1	GGCGACAAGGAGGATAAGGA
ChPINc_qRev1	GGAGTGACTGTTGTGGCAAG
Cloning for *Arabidopsis thaliana*	
CharaPINa_fwd	agcaggcttcCGCGCCATGAAGTGGAAC
CharaPINa _rev	aagctgggtaCTAGTTCACACCAGGAGCTC
CharaPINc_fwd	agcaggcttcCGCGCCATGGAGGTCAAG
CharaPINc_rev	aagctgggtaCTAGTTCACAGAATCTTTTCCAGAATAACG
Cloning for *Marchantia polymorpha*	
(pE2B)eGFPLti6b-F	gtcgactggatccggtaccgATGGTGAGCAAGGGCGAGGAGCTG
eGFPLti6b(pE2B)-R	atatctcgagtgcggccgcgTCAAAAGGTGATGATATAAAGAGC

New Phytologist

Table S3 PIN-FORMED auxin efflux carriers in *Chara braunii.* Designated PIN protein names and corresponding GenBank and UniProt accessions for protein, gene, and scaffold.

**Designated name**	**GenBank protein accession**	**UniProt protein accession**	**GenBank gene accession**	**GenBank scaffold**
CbPINa*	GBG79698	A0A388LBL6	g29962	BFEA01000325.1
CbPINb	GBG79697	A0A388LBJ4	g29961	BFEA01000325.1
CbPINc	GBG64280	A0A388K2K4	g41200	BFEA01000048.1
CbPINd	GBG90244	A0A388M6M8	g50423	BFEA01000798.1
	GBG90245	A0A388M6W5		
CbPINe	GBG90247	A0A388M6M5	g50425	BFEA01000798.1
CbPINf	GBG42734	A0A388JJV1	g84230	BFEA01006036.1

*An updated full nucleotide sequence of CbPINa used for this study is deposited in GenBank with accession number PQ421084.

New Phytologist

Table S4 Estimated affinities of the best-scoring docked ligand poses towards tested receptor.

**Protein**	**Model**	**Affinity / (kcal * mol^-1)**		
		IAA	NAA	NPA
AtPIN3	7WKW	-7,2	-8,1	-9,6
AtPIN3	7XXB	-7,5	-8,2	-9,8
CbPINa	AlphaFold	-6,9	-7,3	-7,8
CbPINc	AlphaFold	-6,7	-7,9	-7,2

Table S5 Significantly hyperphosphorylated proteins under IAA treatment compared to DMSO.

UniProt protein accession	Protein name	Fold Change (Log2)	FDR	Position
A0A388KUQ6	Uncharacterized protein	3,39084	1,8647	988
A0A388MCN6	Sodium/hydrogen exchanger	3,34485	3,9272	502
A0A388MER3	DUF4283 domain-containing protein	3,23836	2,8414	282
A0A388KL36	Uncharacterized protein	3,18321	5,1692	754
A0A388KWQ6	Reverse transcriptase domain-containing protein	3,12867	1,6476	3374
A0A388JYD9	Eukaryotic translation initiation factor 3 subunit B	2,9919	2,1406	6
A0A388KWF0	TATA-binding protein interacting (TIP20) domain-containing protein	2,9870	1,6313	2
A0A388MCN6	Sodium/hydrogen exchanger	2,8504	2,2132	499
A0A388JT36	K Homology domain-containing protein	2,5037	1,3836	2128
A0A388K1B5	Helicase ATP-binding domain-containing protein	2,2454	1,3621	8
A0A388LES0	Calponin-homology (CH) domain-containing protein	2,2139	1,6627	2288
A0A388KF83	Nucleotide-diphospho-sugar transferase domain-containing protein	2,1229	1,9603	18
A0A388L567	Oleosin	1,9048	3,1299	153
A0A388LJZ4	C3HC-type domain-containing protein	1,8777	1,4175	328
A0A388LJZ4	C3HC-type domain-containing protein	1,8699	1,4400	332
A0A388KCH6	Protein kinase domain-containing protein	1,8601	2,9262	1442
A0A388L7R3	BUD13 homolog	1,7689	1,7364	363
A0A388K496	Arf-GAP domain-containing protein	1,7661	1,8030	374
A0A388LX59	DCD domain-containing protein	1,7638	1,5402	391
A0A388JYD9	Eukaryotic translation initiation factor 3 subunit B	1,7616	1,4501	9
A0A388JYD9	Eukaryotic translation initiation factor 3 subunit B	1,7616	1,4501	13
A0A388M738	Lysin motif receptor-like kinase (LysM-RLK)	1,6839	1,3852	366
A0A388M2N1	Cation/H+ exchanger domain-containing protein	1,6712	1,7008	1443
A0A388L963	Integrase catalytic domain-containing protein	1,6210	3,1593	304
A0A388K0S2	Protein kinase domain-containing protein	1,5817	2,4890	1026
A0A388KWB7	Glyceraldehyde-3-phosphate dehydrogenase	1,3011	0,4009	254
A0A388K9B6	CCHC-type domain-containing protein	1,1622	1,9016	246
A0A388KXT1	Uncharacterized protein	1,0705	1,5940	698
A0A388LPE0	Uncharacterized protein	0,9805	1,5237	138
**A0A388LCC7**	Protein kinase domain-containing protein	0,9516	1,3968	685

New Phytologist

Table S6 Significantly hypophosphorylated proteins under IAA treatment compared to DMSO.

UniProt protein accession	Protein name	Fold Change (Log2)	FDR	Position
A0A388LCC7	Protein kinase domain-containing protein	-5,2696	7,8855	793
A0A388LCC7	Protein kinase domain-containing protein	-4,9838	2,1019	332
A0A388LT00	Uncharacterized protein	-4,2944	2,2694	66
A0A388KW32	Uncharacterized protein	-4,1707	1,7532	123
A0A388LLH0	FYVE-type domain-containing protein	-3,7731	1,3609	96
A0A388L139	Methyltransferase small domain-containing protein	-3,7573	4,4580	39
A0A388KV70	PPIase cyclophilin-type domain-containing protein	-3,4496	1,5472	347
A0A388KEQ9	Uncharacterized protein	-3,4427	1,5246	1143
A0A388K086	Calpain catalytic domain-containing protein	-3,2605	1,3025	1662
A0A388KVW7	GPI transamidase subunit PIG-U	-3,1982	1,4446	429
A0A388KH38	1-phosphatidylinositol-3-phosphate 5-kinase	-3,004	3,2468	1919
A0A388L8I5	Uncharacterized protein	-2,9972	1,6645	149
A0A388K5K4	UBX domain-containing protein	-2,6527	1,6661	269
A0A388KT33	mRNA decay factor PAT1 domain-containing protein	-2,593	2,4643	481
A0A388KTB5	Outer arm dynein light chain 1	-2,5618	3,1413	783
A0A388KZ99	Vacuolar protein sorting-associated protein 11	-2,5134	2,1653	420
A0A388KE37	Uncharacterized protein	-2,4793	2,5169	2464
A0A388KD78	Mannan endo-1,4-beta-mannosidase	-2,4221	1,4895	5
A0A388LPG9	ORM1-like protein 3	-2,2788	1,3649	72
A0A388LVU8	Uncharacterized protein	-2,2172	1,5727	328
A0A388JJL4	GAT domain-containing protein (Fragment)	-2,1995	1,3237	219
A0A388K510	DNA-repair protein Xrcc1 N-terminal domain-containing protein	-2,1694	1,5528	709
A0A388KLS2	SEC7 domain-containing protein	-2,1459	1,8358	441
A0A388L3H9	RRM domain-containing protein	-2,0418	1,6903	44
A0A388K510	DNA-repair protein Xrcc1 N-terminal domain-containing protein	-1,894	1,4811	705
A0A388M724	Uncharacterized protein (Fragment)	-1,7887	1,4207	123
A0A388KE37	Uncharacterized protein	-1,7734	1,7555	2859
A0A388L2U2	Integrase catalytic domain-containing protein	-1,7718	1,4357	1566
A0A388L5N4	Coatomer subunit beta'-2	-1,6662	1,5293	852
A0A388JRY3	DUF2252 domain-containing protein	-1,6415	1,6969	474

Methods S1 Construction of cultivation box for *Chara braunii*

A commercial thermoelectric cooler (70 L of inner space) was equipped with a custom LED panel (Fig. S1b; composed of RGB, white, UV, plant growth, and aquarium and UV LED types giving individual powers 8.64, 18.00, 4.32, 7.20 and 7.20 W respectively). The spectrum of each LED was measured by STS VIS spectrometer (OceanOptics, slit 25 µm, Fig. S1c). The intensity of each LED type was fully adjustable via Pulse Width Modulation (PWM) control, managed by a Raspberry Pi microcontroller running a Python script available on https://github.com/vosolsob/Cultivation_box. The PWM signal was generated using the pigpio Python module (https://pypi.org/project/pigpio/), which enables hardware timing on the Raspberry Pi's GPIO output interface, resulting in a smoother PWM signal (eliminating LED flicker) compared to a simple software-based approach. The LEDs were powered by a 12V output from a standard ATX PC power supply via N-channel MOSFETs mounted on a custom-made PCB. An internal 80W Peltier element (TEC1-127080S) was powered by the same supply through a Raspberry Pi-controlled N-channel MOSFET and a two-channel relay configured as an H-bridge, allowing for the repolarization of the Peltier element and enabling both heating and cooling modes. The temperature inside the cooler was monitored by two DHT22 sensors placed in opposite corners, with data fed into the Raspberry Pi. The control script operates in an infinite loop, checking the internal temperature every five seconds. The illumination intensity is regulated according to initial parameters such as 'sunrise' and 'sunset' times and the maximum intensity of each LED type. Illumination can be set to remain constant throughout the day or adjusted according to a sinusoidal curve with a 24-hour period. Temperature regulation can either be constant or variable, depending on a three-point setup ('sunrise', 'maximum', and 'sunset' temperatures). At night, the temperature decreases linearly, while during the day, a combination of linear increase between morning and evening points and a sinusoidal component with a half-period equal to daytime is applied. The maximum temperature of this composite curve is adjusted iteratively to match the desired peak temperature, which is typically reached during the cooler's 'afternoon' hours, mimicking natural conditions. The convergence of the actual temperature to the desired value is achieved by gradually adjusting the power of the Peltier element (through MOSFET regulation by PWM) or its repolarization via

the H-bridge. While this method of Peltier element regulation using pure PWM without an inductance filter is straightforward to implement, it is not optimal. The element is powered at the maximum voltage during the duty cycle, resulting in greater power losses compared to applying a smoothed voltage. Additionally, if the system is powered by 12V, the maximum power of the element is slightly reduced due to a voltage drop across the MOSFET. Depending on the temperature regime, the relay in the H-bridge may experience varying degrees of stress, as observed in our system after two years of continuous operation. This issue could be addressed either through software modifications (e.g., brief shutdown of the MOSFET during H-bridge repolarization) or by implementing a semiconductor-based H-bridge. Several levels of security have been implemented in our system. The first level is integrated into the Python script, where an exceedance of 28°C automatically shuts off the LEDs, and a drop below 2°C turns off the Peltier element. The second level of security is at the operating system level: upon startup (or accidental reboot) of the Raspberry Pi, all GPIO ports are automatically set to zero. A monitoring Bash script also runs, checking every minute whether the main Python script is active; if not, it is restarted with parameters loaded from a configuration file. This automation ensures that the control script does not require manual activation when the cooler is powered on. User control of the system is facilitated through a touch display, allowing full access to the Raspberry Pi's operating system.

Methods S2 Immunolocalization of internodal cells with CbPINs and H^+ATPase

The thalli of young, elongating *Chara braunii*, strain S276, containing several nodes were cut from its media using scissors and fixed in 1% glutaraldehyde in phosphate-buffered saline (PBS; 140 mM NaCl, 2.95 mM KCl, 2.38 mM KH_2PO_4, 7.61 mM Na_2HPO_4, pH 6.9) for 30 min. Fixed internodal cells of thalli were dissected with a scalpel into fragments 3mm long and, separated into acidic and alkaline segments, and further processed as follows: 3x15 min wash with PBS, 30 min treatment with 1 mg ml^{-1} $NaBH_4$ in PBS, 3x15 min wash with PBS. Samples were blocked in 1% (w/v) bovine serum albumin (BSA) and 50 mM glycine in PBS. Primary rabbit antibody against H^+-ATPase was used at a dilution of 1:1000 (AS07260; Agrisera). Polyclonal antibodies against *Chara* PINa and c, raised in rat against epitopes specific to each PIN protein (Moravian Biotechnology, Brno, Czech Republic) were used in dilution 1:500. Primary antibodies were

incubated overnight at 4°C. The next day samples were washed 3x15 min with PBS followed by incubation with secondary antibody, goat anti-Rat IgG (H&L) - Alexa Fluor 488 (A-11006 Invitrogen) for PINs and goat anti-Rabbit IgG (H&L) - Alexa Fluor 546 (A-11035, Invitrogen) for H^+-ATPase in 1% (w/v) BSA and 50 mM glycine in PBS for 2h. The samples were washed 3x30 min wash with PBS. The last wash was done in sterile water for 10 min, after which samples were placed in 50% glycerol. All steps were performed at the room temperature unless specified otherwise.

Methods S3 Immunolocalization of antheridia CbPINs and H^+ATPase

The apical nodes of *Chara braunii* S276 containing generative organs were cut from the rest of the thallus. Cut segments were briefly washed in distilled water that was followed by fixation in 4% paraformaldehyde solution in PBS (pH 7.0) for 45 min with the addition of 0.5 mM $CaCl_2$. Samples were then washed 3x5 with PBS. After that, samples were permeabilized for 10 min in MTSB (50 mM PIPES, 5 mM EGTA, 5 mM $MgSO_4$, pH 7.0; Sigma) containing glycerol (10 %) and Triton X-100 (0.2 %), followed by washing step for 3x5 in MTSB. Samples were treated in ice-cold methanol (-20°C) for 2 min and rehydrated again for 1 min in MTSB. Then cell wall digestion was performed by using 0.1% pectinase from *Aspergillus niger* (Fluka) and 0.01% pectolyase Y-23 (ICN) for 15 min in MTSB which was followed by washing 3x5 in MTSB. Samples were then permeabilized a second time in 10% (v/v) DMSO and 3% (v/v) Nonidet P-40 in MTSB for 1h. After washing 3x5 in MTSB, the samples were placed on superfrost glass (Epredia™ SuperFrost Plus™). The antheridia were dissected from thallus under a binocular microscope, squashed to release the antheridial filaments and air-dried. The slides were then blocked for 1h with 1% BSA in MTSB. The slides were then incubated with a primary antibody containing 1% BSA in MTSB in a humid chamber overnight at 4°C and washed 3x5 with MTSB the next day, followed by incubation with secondary antibody for 2h. The same primary and secondary antibodies were used as in the protocol for immunolocalization of internodal cells (as described above). The samples were washed 3x5 in MTSB. In the last washing step, DAPI was added in a concentration of 1 µg ml^{-1}. The samples were then washed with sterile water and placed in 50% glycerol.

Methods S4 Immunolocalization of tubulin in internodal cells

For the immunostaining of microtubules, thalli were pretreated with perfusion solution (200 mM sucrose, 70 mM KCl, 4.49 mM $MgCl_2$. 5 mM EGTA, 10 mM PIPES, pH=7. followed by fixation for 10 min with 1% glutaraldehyde in PBS and cutting off the nodes. After washing in PBS, thalli segments were incubated with 50 mM glycine in PBS and blocked with 1% BSA in PBS for 1h. Primary monoclonal antibodies against α-tubulin (DM1A) (1:1000) in PBS were incubated overnight. The following day, samples were incubated with a secondary, goat anti-mouse IgG, Alexa Fluor™ 488 (A28175), (1:1000) for 3h, after which they were washed with PBS. The final wash was done using a 200 mM sucrose solution.

Methods S5 Immunolocalization of tubulin in antheridial cells

Thallus tips containing antheridia were incubated for 1h in a 5 µM taxol solution. Then, the samples were fixed for 40 min in a 1% glutaraldehyde in PMET buffer (0.05% Triton X-100, 50 mM PIPES, 5 mM EGTA, 1 mM $MnSO_4xH_2O$, pH 7.2). After fixation, the samples were washed 3x for 10 min in PMET buffer, followed by cell wall digestion (0.1 % pectinase from *Aspergillus niger* (Fluka) and 0.01 % pectolyase Y-23 (ICN) for 20 min. After washing the samples were then incubated in a permeabilization buffer for 3h at room temperature and then rinsed three times for 10 min in PBS. Following the washing, samples were incubated with a blocking solution containing 1% BSA in PBS for 60 min. The primary and secondary antibodies step was the same as in internodal cells. Just before the final wash, the samples were stained with 1 µg ml^{-1} Hoechst 33342 for 5 minutes.

Methods S6 Protein extraction and Western blot

Around 500 mg of fresh *Chara braunii* thalli strain S276, including rhizoids, was harvested, washed in distilled water, and briefly blotted with a paper towel to remove the excess water. Thalli were homogenized in precooled mortar and pestle with liquid nitrogen. The homogenate was transferred to cold 15 mL centrifuge tube, resuspended in a cold extraction buffer (330 mM saccharose, 100 mM KCl, 1 mM EDTA, 50 mM Tris–HCl, pH 7.4), 0.5 mM phenylmethylsulfonyl fluoride (PMSF), 5 mM dithiothreitol (DTT) and 1% (v/v) protease inhibitor cocktail (P9599;

Sigma) at a ratio of 0.5 ml g^{-1} FW. Cell debris was removed by centrifugation at 3,000xg for 10 min at 4°C. The pellet was discarded while the supernatant was centrifuged at 100,000xg for 1h at 4°C. The resulting pellet representing the solubilized membrane fraction was resuspended in the extraction buffer. Protein extracts were mixed in ratio 1:1 with 2D buffer, then separated on a 10% SDS gel electrophoresis. SDS gels were transferred to a nitrocellulose membrane by electro-blotting (Trans-Blot® Turbo™ Transfer System, BioRad). Western blots were probed with rat anti-CbPINa (1:1000), rat anti-CbPINc (1:1000), rabbit anti-AHA (1:2000) and mouse anti-α-tubulin as positive controls, rat pre-immune serum (1:1000) as a negative control, and respective secondary HRP-conjugated antibodies (rabbit anti-rat HRP conjugate ENZO ADI-SAB-200-J 1:5000, goat anti-rabbit HRP conjugate, ENZO ADI-SAB-300-J 1:5,000) Proteins were visualized using the enhanced chemiluminescence (ECL) method (Pierce Western Blotting Substrate) and Azure 600 Imaging System.

Methods S7 Sample preparation for phosphoproteomic analysis

Plant material was harvested, frozen in liquid nitrogen, ground to a fine powder, and stored at -80°C until further processing. For comparisons between treatments, all replicates of all treatments were grown on the same day and processed independently.

Methods S8 Protein extraction for phosphoproteomic analysis

For protein extraction, samples were suspended in an extraction buffer with 100 mM Tris-HCl pH 8.0, 7 M Urea, 1% Triton-X, 10 mM DTT, 10 U/ml DNase I (Roche), 1 mM $MgCl_2$ and 1% benzonase (Novagen) and lysed by sonication using 30 cycles of 30 seconds ON and 30 seconds OFF at 90% amplitude at 4°C using a waterbath sonicator (Qsonica). Lysate was cleared by centrifugation at 20.000xg for 30 minutes at 4°C. The supernatant was collected and an extra 1% (v:v) of benzonase was added and incubated for 30 minutes at room temperature. Followed by alkylation in 50 mM Acrylamide for another 30 minutes at room temperature. After alkylation, proteins were precipitated using methanol/chloroform. To the one volume lysate, methanol, chloroform, and milliQ were added in a ratio 4:1:3 with rigorous vortexing between each addition. Lysate was centrifuged for 10 minutes at 5000xg. After centrifugation, the top layer was discarded, and 3 volumes of methanol were added to precipitate the protein layer by

centrifugation for 10 minutes at 5000xg. After centrifugation, the supernatant was discarded, and the protein pellet was air-dried. Next, protein pellets were resuspended in 50 mM ammonium bicarbonate (ABC) and protein concentration was measured by Bradford reagent (Biorad). For every replicate 500 µg protein was digested overnight at room temperature with sequencing grade trypsin (Roche) in a ratio of 1:100 trypsin:protein. After digestion, peptides were desalted and concentrated using homemade C18 microcolumns. Microcolumns were produced using disposable 1000 µl pipette tips that were fitted with 4 plugs of C18 octadecyl 47 mm Disks 2215 (Empore™) material and 1 mg:10 µg of LiChroprep® RP-18 (Merck): peptides. Microcolumns were sequentially washed with 100% methanol, 80% Acetonitrile (CAN) in 0.1% formic acid and twice equilibrated with 5% Acetonitrile in 0.1% formic acid. These steps were performed by centrifugation for 2 minutes at 1500xg. Peptides were loaded onto equilibrated columns for 30 minutes at 400xg. Bound peptides were washed with 5% Acetonitrile in 0.1 % formic acid and eluted with 80% ACN in 0.1% formic acid for 2 minutes at 1500xg.

Methods S9 Phosphopeptide enrichment

Phospho-peptide enrichment was performed using PureCube Fe-NTA MagBeads magnetic beads (Cube Biotech) following the manufacturer's instructions. Eluted peptides were acidified using 10% formic acid. Acidified samples were desalted and concentrated using homemade C18 microcolumns. Microcolumns were produced by fitting disposable 200 µl pipette tips with 2 plugs of C18 octadecyl 47 mm Disks 2215 (Empore™) material and 1mg:10 µg of LiChroprep® RP-18 (Merck): peptides. Microcolumns were washed and equilibrated as described above. Peptides were loaded onto equilibrated microcolumns for 30 minutes at 400xg, 5% acetonitrile in 0.1% formic acid, and eluted with 80% ACN in 0.1% formic acid for 2 min at 1500xg. Eluted peptides were subsequently concentrated using a vacuum concentrator for 30-60 minutes at 45°C and resuspended in 15 µl of 0.1% formic acid.

Methods S10 Statistical analysis

For *Chara braunii* regeneration experiments the thallus length and side branch numbers were measured after 15 days and analyzed using GLMM in the lme4 package (Bates *et al.,* 2015). Gamma and Poisson distributions were used for shoot lengths and numbers, respectively.

Replicates from three independent experiments were modeled as a random effect in the linear mixed-effects analysis. Treatment significance was assessed by likelihood-ratio test, and differences between treatments were analyzed using 95% confidence intervals with the emmeans package (Searle *et al.,* 1980).

For cytoplasmic streaming full-length movies (1 min) were converted into individual images (2 frames per second), and particles in the last 10 seconds of each movie were tracked manually using Fiji imaging software. For each treatment, 10 particles per cell were analyzed across 3 individual cells, with three independent replicates of the treatment. The median velocity for each particle was determined, and a linear mixed-effects model (lmer) was fitted to the log-transformed velocities, incorporating nested random effects for replicate and cell identity. Two controls with different amounts of DMSO were merged together. The significance of a single fixed effect, representing both the treatment type and concentration, was assessed using a likelihood-ratio test. Estimated marginal means (emmeans) were calculated to identify differences between groups.

For gravitropic bending assay each variant, length and direction of the resultant vector were calculated using package 'circular' in R and tested by estimation of 95% confidence intervals based on non-parametric bootstrapping (with 1000 replications) of original measurements and 1000 randomly generated sets of directions of the same length as original measurements (N = 30).

For auxin transport assays the whole experiment with orthogonal design of induction and NPA treatment was done in triplicates and repeated two times. Asymptotic growth curves (SSasymp) of the tracer accumulation were fitted by nonlinear least-square model ('nls' in R) to individual replications and parameters of asymptotic growth curves were obtained. The parameter describing the asymptotic concentration was fitted by linear mixed-effects model (lme4::lmer) introducing replicates as random effects and estradiol induction and NPA treatments as fixed effects. The significance were tested by $\chi 2$-test and Individual differences were tested by the emmeans::pairs function.

For phosphoproteomic analysis data was imported to Perseus and filtered for reverse and potential contaminants. Phosphosite localization probability was filtered using a cut-off

≥0.75. Intensity values were log2 transformed and filtered to contain at least 75% valid values in each group in at least one condition. Values were subsequently normalized by median column subtraction and missing values were imputed from a normal distribution using standard settings in Perseus (width: 0.3, down shift: 1.8). FDR permutation-based t-tests were done in pairwise comparisons (IAA versus DMSO, BA versus DMSO and IAA versus BA). Phosphosites passing the cut -off (*FDR* ≤0.05) were further analyzed. Adobe Illustrator and R, using standard packages, were used for data visualization.

Video S1 Cytoplasmic streaming of branchlet internodal cells after DMSO treatment.

Video S2 Cytoplasmic streaming of branchlet internodal cells after 0.1 µM IAA.

Video S3 Cytoplasmic streaming of branchlet internodal cells after 1 µM IAA.

Video S4 Cytoplasmic streaming of branchlet internodal cells after 0.1 µM BA.

Video S5 Cytoplasmic streaming of branchlet internodal cells after 1 µM BA.

(videos attached as separate files)

References

Bates D, Mächler M, Bolker B, Walker S. 2015. Fitting Linear Mixed-Effects Models Using lme4. *Journal of Statistical Software* 67: 1–48.

Searle SR, Speed FM, Milliken GA. **1980**. Population Marginal Means in the Linear Model: An Alternative to Least Squares Means. *The American Statistician* **34**: 216–221.
